# Design Considerations for Essential Oil Formulations: Emulsification, Encapsulation and Stabilisation Strategies

**DOI:** 10.3390/pharmaceutics18080953

**Published:** 2026-08-01

**Authors:** Sophie E. Whyms, Helen Sheridan, John J. Walsh

**Affiliations:** 1 NatPro Centre, School of Pharmacy and Pharmaceutical Sciences, Trinity College Dublin, D02 PN40 Dublin, Ireland; whymss@tcd.ie (S.E.W.); hsheridn@tcd.ie (H.S.); 2 School of Pharmacy and Pharmaceutical Sciences, Trinity College Dublin, D02 PN40 Dublin, Ireland; 3 Faculty of Pharmacy, Biruni University, Istanbul TR-34015, Türkiye

**Keywords:** essential oils, encapsulation, emulsification, formulation design, molecular interactions, cinnamon, lavender, thyme, rosemary, terpene compounds

## Abstract

Essential oils are widely recognised for their bioactive properties and therapeutic effects. Despite their potential, their practical application is often limited by intrinsic physicochemical constraints, including volatility, chemical instability, hydrophobicity and, most of all, compositional complexity. The development of effective delivery systems is therefore key to stabilise essential oil constituents, improve bioavailability and enable controlled release. Here we provide a comprehensive analysis of formulation strategies for essential oils, focusing on emulsification and encapsulation techniques. Emulsion-based delivery systems, including nanoemulsions and microemulsions, are discussed in relation to their interfacial behaviour, surfactant interactions and preparation methods. In parallel, encapsulation systems, such as vesicular systems, lipid-based carriers, polymeric nanoparticles and inclusion complexes, are evaluated according to their structural characteristics, protective capabilities, encapsulation efficiencies and release profiles. Collectively, these approaches are examined in the context of their influence on the stability, functionality, and overall performance of essential oil formulations. Emphasis is placed on essential oil-specific challenges that arise when preparing a formulation, especially the heterogenous nature of their chemical composition and its impact on physicochemical properties, partitioning, loading capacity, stability and safety. To contextualise these principles, representative examples of essential oil-based formulations (e.g., thyme, clove, rosemary and mint), formulated singular metabolites (e.g., carvacrol, eucalyptol, limonene and eugenol) and essential oil patent applications (>20,000) and products are outlined to illustrate behaviour and performance within the respective delivery system. Overall, the fundamental design considerations that guide the rational selection, preparation and evaluation of formulations for essential oil-based applications are discussed. Considerations for future directions are also outlined.

## 1. Introduction

Essential oils (EOs) are complex, volatile, aromatic mixtures of secondary metabolites biosynthesised by plants, traditionally recognised for their organoleptic, therapeutic and bioactive properties. Over the past several decades, EOs have attracted scientific and industrial interest across diverse domains, including pharmaceuticals, nutraceuticals, cosmetics, food preservation, antimicrobial applications and, increasingly, advanced biomedical delivery systems. Their multifaceted bioactivities, including anti-inflammatory, antioxidant, anticancer, insect repellent, and analgesic properties, to name a few, are attributed to the diverse profiles of terpenes and other low-molecular-weight constituents, including, in certain instances, the phenolics that make up EOs [1,2]. Despite strong evidence of efficacy in both in vivo and in vitro contexts, translating the intrinsic biological potential of EOs into clinically and commercially viable products has historically faced significant challenges. EOs that have not undergone any incorporation into a matrix tend to evaporate rapidly, degrade under environmental stressors and exhibit limited bioavailability when administered topically or systemically [3]. The development of sophisticated formulations for EOs is not merely a surface-level exercise in dilution or carrier oil blending but an integrated approach that combines analytical and polymer chemistry, colloid science, pharmaceutical technology and an understanding of biological barriers to delivering the desired outcomes [2,3]. An effective formulation strategy must address multiple, often competing objectives, such as improving the stability of volatile components, controlling the release profile over desired durations, enhancing solubility and bio-accessibility, minimising irritation or toxicity and, above all, successfully incorporating the whole oil or desired bioactive constituents for a specific application, e.g., biomedical, cosmetical, agricultural, etc. However, despite their broad potential, the translation of EO-based technologies remains limited by their inherent instability, which can reduce efficacy and result in inconsistent performance and shortened shelf life, thus representing major barriers to regulatory approval and commercial adoption. These challenges are particularly significant when transitioning from laboratory-scale demonstrations to reproducible, scalable products, where maintaining constituent stability, controlling release and ensuring safety become critical considerations. The economic implications of these limitations are considerable, as instability during storage, transport and application can reduce product efficacy and increase manufacturing complexity and costs.

**Figure 1:**
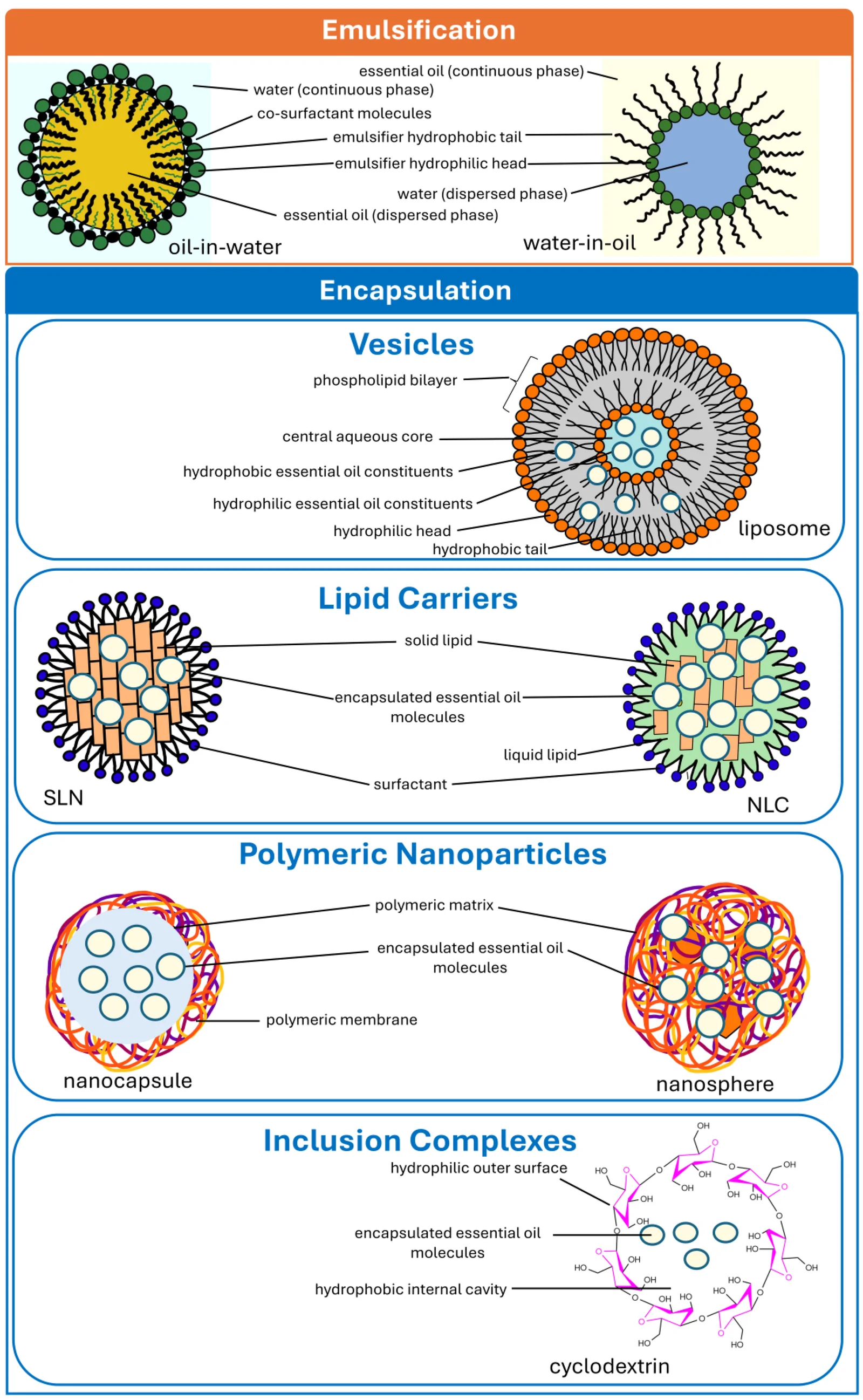
Formulation strategies for EOs.

Achieving these goals has driven the innovation of diverse formulation classes, including emulsions (nano and micro) [4] and encapsulation systems such as vesicles, lipid carriers [5,6], polymeric nanoparticles (PNs) [7] and inclusion complexes (ICs) (Figure 1) [8]. Over the past two decades, considerable research has focused on adapting and optimising these platforms for EOs and their components. In food systems, EO nanoemulsions and encapsulated carriers have demonstrated antimicrobial efficacy against foodborne pathogens and are now incorporated into edible coatings and active packaging to extend shelf life [9,10]. In cosmetic and dermatological contexts, liposomal and lipid carrier formulations of EOs such as *Lavandula angustifolia* Mill. (lavender) and *Mentha* × *piperita* L. (peppermint) have improved skin penetration, wound healing and anti-ageing performance [2]. In pharmaceutical applications, polymeric and vesicular EO nanoformulations are being explored for targeted antimicrobial therapy, anticancer delivery and wound care [1,11,12]. In agriculture, ICs and chitosan-based systems provide controlled biopesticide EO release, reducing environmental loss and extending field efficacy [13]. Not only in a research context but in the commercial landscape as well, EO-based formulations are no longer an emerging niche market but a rapidly developing sector, spanning agriculture, food preservation, cosmetics, household products and pharmaceutics. Patent activity over the last several years has concentrated on advanced delivery platforms such as those discussed in this review, reflecting continued industrial investment in translating laboratory efficacy into commercially viable products. Beyond a significant number of patents in the application stage (>20,000) [14,15,16,17,18,19,20,21,22], there is notable evidence of successful commercialisation within several sectors, with agriculture being a primary example. Mevalone^®^, a terpene-based (geraniol, and thymol) biofungicide using Eden Research’s Sustaine^®^ encapsulation technology [23] and Cedroz^®^ by Eastman, another terpene-based (geraniol, thymol) bioematicide, are both registered across multiple jurisdictions, including Europe and North America, demonstrating regulatory acceptance of formulation EO actives [24]. At a whole EO level, Ecotrol^®^ by KeyPlex, containing *Rosmarinus officinalis* L. (rosemary) and (peppermint) EO, has been commercialised as a botanical insecticide. Furthermore, formulation technologies based on CDs, liposomes, nanoemulsions and lipid carriers have generated numerous patents and commercial ingredients for cosmetic, personal-care and food-preservation applications [25,26]. Products such as CelluOil^®^ by Tagra, microcapsules containing menthol [27], and RootBioTec™ by Mibelle Biochemistry, containing *Ocimum basilicum* L. (basil) EO, are marketed to consumers interested in personal care and cosmetics [28]. Collectively, the combination of sustained patent generation, continuing product registrations and successful commercial examples demonstrates that EO formulation research is a growing and commercially relevant area. New encapsulation technologies are continually being developed, with approaches such as porous carriers, including metal-organic frameworks (MOFs) [29,30], and mesoporous silica materials such as MCM-41 [31], MCM-48 [32] and SBA-15 [33]. Their high surface area, adaptable pore structure and high loading capacity can improve EO stability while enabling sustained release, making them promising candidates for future pharmaceutical and food packaging applications [34,35,36].

The literature for this review was identified through searches on the PubMed, Scopus and Web of Science databases. Additional relevant publications were identified by screening the reference lists of retrieved articles and through searches of Google Scholar, where appropriate. Searches focused predominantly on the literature between 2000 and 2026 using combinations of keywords, including essential oil, encapsulation, formulation, nanoemulsion, microemulsion, liposome, solid lipid nanoparticle, nanostructure lipid carrier, polymeric nanoparticle, cyclodextrin, inclusion complex, controlled release, drug delivery, intermolecular interactions, stability, physicochemical properties and terpene. Original research articles, review articles and, where relevant, patents and commercially available technologies were included to provide a comprehensive overview of formulation strategies for EOs (Figure 1).

## 2. Definition and Classification

### 2.1. Emulsification-Based Delivery Systems

Emulsions, including nanoemulsions and microemulsions, are colloidal systems formed by the dispersion of one immiscible liquid phase within another, typically stabilised by surfactants [4]. Emulsions can be broadly classified as oil-in-water (O/W), where oil droplets are dispersed in a continuous aqueous phase, or water-in-oil (W/O), where water droplets are dispersed in oil [37]. The two colloidal systems, micro- and nanoemulsions, are frequently grouped together due to their small droplet size and overlap in reported size ranges; however, they differ fundamentally in their thermodynamic properties [38,39].

Microemulsions are thermodynamically stable systems that form spontaneously when oil, water, surfactant and, sometimes, a co-surfactant are mixed in appropriate ratios [4,39]. These phases may be water-continuous, oil-continuous or bi-continuous, dependent on the concentrations, nature and arrangement of the molecules present in the mixture. O/W microemulsions represent the most common approach for EO emulsification and typically form spheroid structures of the oil and surfactant within the aqueous phase [39]. This is a result of the surfactant’s non-polar tail association leading to the creation of a hydrophobic core. When considering EOs in O/W microemulsions, the presence of EO constituents with functional polar groups may lead to a deviation in shape from spheroid to cylindrical. Amphiphilic terpenes such as linalool will orientate themselves so that the hydrophobic part of the molecule will sit inside the core, while the polar portion will point towards the interface, interacting with the hydrophilic surfactant molecule heads or even partially with the aqueous medium [40].

Nanoemulsions are distinguished from microemulsions, as they are kinetically stable systems rather than thermodynamically stable and require the input of external energy in order to form [39]. Their long-term stability depends on maintaining small droplet sizes and preventing coalescence or ‘Ostwald ripening’ [41]. The use of surfactants is not necessary; however, they are often used to facilitate formation and ensure kinetic stability during storage [39]. In practice, EO nanoemulsions have achieved regulatory-accepted use in food flavouring and are increasingly deployed in active packaging systems, edible coatings for food and cosmetic emulgels for topical skin delivery as well minor applications in pest and crop management [9,10,42,43,44,45,46].

### 2.2. Encapsulation-Based Delivery Systems

Encapsulation refers to a broad set of delivery technologies designed to physically entrap active molecules within a carrier matrix or structure, with the dual aims of protecting labile compounds from degradation, oxidation and evaporation, as well as modulating their release into the target environment [47,48,49]. Encapsulation systems can be broadly classified based on their structural and compositional characteristics:

- Vesicular systems (liposomes, niosomes, ethosomes).
- Polymeric nanoparticles (PNs) (chitosan, poly(lactic-co-glycolic) acid (PLGA)-based systems).
- Lipid carriers, such as solid lipid nanoparticles (SLNs) and nanostructured lipid carriers (NLCs).
- Inclusion complexes (ICs) (cyclodextrins (CDs)).

These categories cover the principal strategies explored by EO encapsulation research, each with unique advantages and limitations in terms of incorporation, release behaviour and scalability [48].

#### 2.2.1. Vesicular Encapsulation Systems

Vesicles are spherical structures comprising amphiphilic lipids or surfactant bilayers that self-assemble in an aqueous medium [48]. Liposomes are the most extensively studied for EO delivery over ethosomes and niosomes due to their biocompatibility, structural resemblance to biological membranes and ability to encapsulate both hydrophilic and hydrophobic molecules within the lipid bilayer [48,50]. Depending on their lamellarity and size, liposomes can be classified as small unilamellar vesicles (SUVs) 20–100 nm, large unilamellar vesicles (LUVs) > 100 nm, or multilamellar vesicles (MLVs) > 0.5 µm [48]. The size and layering of the vesicles can impact the physical stability and release kinetics of the system. Liposomes are biodegradable, nontoxic, nonimmunogenic and biocompatible and can offer several advantages as carriers for EOs [51]. However, challenges such as bilayer oxidation and leakage need to be addressed in formulation design [52].

#### 2.2.2. Lipid-Based Carrier Systems

SLNs and NLCs are lipid-based nanocarriers formed from solid lipids (SLNs) or a mixture of solid and liquid lipids (NLCs). SLNs are spherical carriers made of crystallised lipid droplets in which hydrophilic or hydrophobic compounds are directly incorporated or dissolved. Unlike vesicular systems, which rely on bilayer self-assembly, SLNs and NLCs consist of a solid or semi-solid lipid core stabilised by surfactants. This leads to the formation of colloidal dispersions, typically in the nanometre size range [47]. In these systems, encapsulation occurs through incorporation of active compounds within the lipid core during the crystallisation process, resulting in physical entrapment within the lipid matrix [53,54]. This structural organisation provides an effective barrier against volatilisation, oxidation and chemical degradation, making the systems a suitable carrier for EOs [55]. The key distinction between SLNs and NLCs lies in the internal structure and lipid composition of the core. SLNs crystallise into highly ordered lattices upon cooling. In contrast, NLCs are a less ordered, imperfect crystalline structure that introduces structural defects within the matrix. These imperfections increase the available space for active compounds, thereby enhancing encapsulation efficiency (EE) [54,55]. Despite the improved release, greater colloidal stability and enhanced bioavailability that SLNs and NLCs offer, the temperatures required (usually 60–90 °C, depending on the lipid melting point) for their preparation may cause degradation to heat sensitive components present in EOs [56,57]. Monoterpenes and other low-boiling EO components are particularly susceptible to thermal loss during homogenisation and emulsification processes. SLN and NLC formulations of EOs, including *Syzygium aromaticum* L. (clove), lavender, *Origanum vulgare* L. (oregano) and *Zataria multiflora* Boiss. (Shirazi thyme), have been successfully applied as antimicrobial food packaging agents and topical dermal vehicles, with NLC systems additionally being investigated for cosmeceutical anti-ageing and skin-barrier applications [1,11,58,59,60].

#### 2.2.3. Polymeric Nanoparticle Systems

These consist of biodegradable polymers such as chitosan, PLGA, polyvinyl alcohol (PVA) and others, which form colloidal particles typically ranging from tens to several hundreds of nanometres. These carriers provide controlled release, targeted delivery potential and enhanced protection against environmental degradation [61]. The polymer matrix may incorporate EOs either by entrapment during nanoparticle formation or by adsorption onto the particle surface. Studies show that polymeric systems can improve the stability and bioactivity of EOs while enabling surface modification for targeted interaction with biological tissues or microbial cells [57,61]. Applications span food preservation, wound healing and agriculture. PLGA-based PNs encapsulating *Cymbopogon nardus* L. (lemongrass) EO have demonstrated enhanced activity against drug-resistant *Pseudomonas aeruginosa* [62], while chitosan nanoparticles containing peppermint EO have been deployed against grain insect pests [63].

#### 2.2.4. Inclusion Complex Systems

These represent a molecular-level encapsulation strategy in which guest molecules are partially or fully entrapped within the cavity of a host compound through non-covalent interactions (e.g., van der Waals forces, hydrogen bonding) [49,64]. In EO delivery systems, the most widely used host molecules are CDs, which are cyclic oligosaccharides that form toroidal structures composed of glucopyranose units. They are characterised by a hydrophilic external surface and relatively hydrophobic internal cavity, suitable for some constituents of EOs [64]. Based on the number of glucose units, the most common CDs are classified as α-CDs (6 units), β-CDs (7 units) and γ-CDs (8 units). Among these, β-CDs and their derivatives, such as hydroxypropyl-β-CDs (HP-β-CDs), are most frequently used due to their cavity size and compatibility with monoterpenes and phenolic compounds [49,64]. ICs are particularly valued in food flavouring, where CD complexation can increase the half-life of volatile terpenoids, mask strong odours and flavours, and enable the slow release of aromatic compounds in functional food systems [65,66]. In agriculture, CD-complexed EOs provide controlled biopesticide release with reduced volatility losses in field conditions [13].

## 3. Physicochemical Principles and Composition

### 3.1. Physicochemical Characteristics of Emulsification Systems

The formulation of emulsions is often facilitated by surfactants that reduce the interfacial tension between the two otherwise immiscible liquids [67]. Surfactants and co-surfactants rapidly adsorb at the oil–water interface during emulsification, forming a stabilising interfacial layer around the droplets that prevents coalescence and aggregation [39,68]. The selection and ratio of surfactants largely determine the physicochemical characteristics and stability of the resulting system. Surfactants are commonly classified according to their hydrophilic–lipophilic balance (HLB), which reflects the relative contributions of the hydrophilic and lipophilic parts of the molecule. For O/W systems containing EOs, surfactants with moderate to high HLB values, i.e., leaning on the more hydrophilic side, are generally required to promote dispersion in water [69]. Nirmal et al. [70] ascertained that the optimum surfactants for *Backhousia citriodora* F. Muell. (lemon myrtle) and *Syzygium anisatum* (Vickery) Craven & Biffin (anise myrtle) EOs, to achieve good droplet size and PDI, were Tween 80 (HLB 15) and Span 80 (HLB 4.3) at various combinations. Non-ionic surfactants like these are more commonly used for EO emulsions due to their low toxicity, chemical stability and regulatory acceptance. Other common examples include sucrose esters and polyoxyethylene ether surfactants [9,71,72]. Natural surfactants such as lecithin, saponins and the newer rhamnolipids as well as ionic surfactants are not typically utilised in EO emulsions as they cannot generate the ultralow interfacial tension required [4] and present cytotoxicity concerns [73].

Many microemulsions and low-energy nanoemulsions also require the presence of a co-surfactant [70]. Co-surfactants are usually small amphiphilic molecules that cannot independently stabilise emulsions but enhance the performance of primary surfactants by further reducing interfacial tension and increasing interfacial film flexibility. These molecules insert between surfactant molecules at the oil–water interface, disrupting head group interactions and improving curvature adaptability of the interfacial layer [4,74]. They are especially important in microemulsions, where ultralow interfacial tension is necessary to achieve thermodynamic stability. Short- and medium-chain alcohols and polyols such as ethanol propylene glycol, glycerol and PEG derivatives are the most commonly used co-surfactants [75]. Ethanol is particularly prevalent in EO-based systems because it promotes the formation of water-dilutable emulsions [76,77]. Furthermore, in some EO systems, individual oil constituents may also contribute to interfacial activity. Compounds such as terpene alcohols and phenolic constituents like eugenol can behave as intrinsic co-surfactants due to their amphiphilic nature, assisting in interfacial stabilisation and potentially facilitating microemulsion formation [78]. EOs typically possess relatively low viscosity and interfacial tension, facilitating droplet disruption during emulsification and enabling the formation of small droplets [4]. However, some EO constituents also exhibit relatively higher water solubility compared to long-chain triglycerides, which makes nanoemulsions containing them particularly susceptible to Ostwald ripening, the dominant destabilisation mechanism of dispersed oil molecules through the aqueous phase from smaller droplets to larger droplets, resulting in progressive droplet growth [4]. To mitigate this phenomenon, highly hydrophobic fixed oils such as medium-chain triglycerides, long-chain triglycerides or vegetable oils are frequently incorporated as ripening inhibitors. The fatty acid composition and degree of unsaturation influence oil hydrophobicity, viscosity and molecular diffusion and therefore slow droplet growth [4]. Formulators assess the overall stability of emulsions by measuring droplet size, polydispersity index (PDI), zeta potential and stability over time. These serve as practical indicators of the underlying interfacial thermodynamic principles.

### 3.2. Physicochemical Characteristics of Encapsulation Systems

In contrast to emulsions where stabilisation occurs primarily at liquid–liquid interfaces, many encapsulation systems involve the formation of discrete molecular or solid-phase structures that physically confine active compounds within a defined carrier architecture; if the bioactive constituent with an EO is known or the EO chemical composition is relatively simple (e.g., *Citrus* L. (lemon), rich in limonene), this can be an extremely useful approach for formulating these oils. These interactions are typically driven by the minimisation of system free energy, hydrophobic effects, electrostatic attraction and steric stabilisation [79,80].

#### 3.2.1. Composition and Physicochemical Principles of Vesicular Systems

Liposomes are vesicular systems governed by principles of spontaneous self-assembly, intermolecular interactions and interfacial thermodynamics. Their formation arises from the amphiphilic molecular structure of phospholipids. When dispersed in water, the system minimises free energy by orientating hydrophobic tails inward, away from the aqueous environment, while hydrophilic head groups remain exposed to water, producing bilayer structures that close to form vesicles [79,81]. The important physicochemical parameters of liposomes, as outlined by the FDA, include physical properties (size, zeta potential, PDI), lamellarity, EE, phase transition temperature, thermal stability, composition of the lipid bilayer and sterility [82,83]. The composition of the lipid bilayer is the primary determinant of liposomal physicochemical behaviour. Phospholipids are based on a glyceryl ester backbone that has a hydrophilic head group and hydrophobic acyl chain attached to it. Phosphatidylcholine (PC) and phosphatidylethanolamine (PE) sourced from eggs and soya beans are amongst the most frequently used glycerophospholipids in EO liposome formulation [48,82]. Differences in the polar head group result in a variety of phospholipid bilayer structures, such as 1,2-dioleoyl-PC (DOPC), forming stable lamellar structures, whereas DOPE forms inverted hexagonal structures [82]. Variations in head group chemistry, acyl chain length and degree of saturation also influence bilayer packing, fluidity and permeability [82]. Phospholipids with long, saturated acyl chains form tightly packed, ordered bilayers with reduced permeability, enhancing the retention of volatile EO components. In contrast, unsaturated lipids introduce kinks in the acyl chains, resulting in more fluid and disordered membranes that may increase the leakage of encapsulated compounds [79,81,82].

The size and number of bilayers directly influence encapsulation behaviour and release characteristics. Unilamellar vesicles contain a single bilayer surrounding an aqueous core, while MLVs consist of multiple concentric bilayers. SUVs typically exhibit greater surface area-to-volume ratios, facilitating more rapid release, whereas MLVs provide enhanced retention of lipophilic compounds within the multiple bilayer domain [81,82]. In EO-loaded liposomes, lipophilic constituents such as terpenes preferentially partition into the hydrophobic bilayer region, where they are stabilised by van der Waals interactions in the lipid acyl chains [84]. Low PDI values (<0.3) indicate uniformity and are associated with improved physical stability and reproducible release profiles. High PDIs can lead to instability through aggregation, fusion or differential release [80,82]. Cholesterol is commonly incorporated into EO-liposomal formulations as a membrane-modifying agent by insertion between phospholipid molecules, reducing bilayer fluidity in the liquid-crystalline phase and preventing excessive rigidity in the gel phase. This dual effect results in enhanced membrane stability and reduced permeability [82]. In EO-loaded liposomes, cholesterol plays a particularly important role in reducing volatilisation and oxidative degradation by limiting diffusion through the bilayer [80].

EE reflects the proportion of active compound successfully incorporated into the liposomal system [82]. Accurate determination of encapsulation requires quantification of at least two of three parameters: total content of a marker compound(s), encapsulated marker compound(s) fraction and concentration of free marker compound(s). It can be calculated as

EE% = (1 − C_f_/C_t_) × 100% (1)

C_t_ = C_f_ + C_e_

where C_t_ is the total amount of drug, C_f_ is the amount of free drug and C_e_ is the amount of encapsulated drug [85]. In liposomes containing EO, this is influenced by lipid composition, preparation method and the physicochemical properties of the metabolites present in the EO, particularly their molecular size and polarity. Release behaviour is governed by diffusion through the bilayer, vesicle permeability and structural integrity. As the liposomes tend to display selective affinity towards some EO components over others, those that embed themselves within the bilayer will show sustained release profiles over those that are loosely associated or not incorporated at all. Due to this, the EE can vary widely for EOs in liposomes, often due to the preparation method and complexity of the EO itself [6,48].

Surface modification strategies are often used to improve liposome stability and functionality. The incorporation of hydrophilic polymers such as PEG produces sterically stabilised liposomes, which exhibit reduced aggregation and prolonged circulation times. This is particularly relevant to EO-loaded liposomes, as EOs can modify the surfaces of vesicles [6,86]. Monoterpenes have been demonstrated to decrease the size of liposomes by forcing soybean PC vesicles to increase the surface curvature. This is likely due to their interactions at the polar head group region of the molecule [48,87]. In addition to surface modification, surface charge, commonly expressed as zeta potential (mV), is a critical factor influencing colloidal stability. Liposomes with high positive or negative zeta potential values exhibit electrostatic repulsion, which prevents aggregation and enhances dispersion stability. Opposingly, neutral liposomes are more prone to flocculation and fusion [48]. Surface charge is governed by the phospholipid head groups and any incorporated charged molecules.

#### 3.2.2. Composition and Physicochemical Principles of Lipid Carriers

SLNs and NLCs are managed by unique principles of lipid crystallisation, interfacial stabilisation and mass transport phenomena [47]. Particle size is typically a good measure of stability for SLNs and NLCs, and it is often used to characterise the final product. An indication of physical instability is if particles are found to be greater than 1 µm, with an increase in their number over time [88].

The physicochemical behaviour of SLNs and NLCs is strongly influenced by lipid composition and crystallinity, which determine the internal organisation of the matrix and the mobility of encapsulated compounds [88,89]. Lipids commonly used include triglycerides, fatty acids and waxes, selected based on their melting point and compatibility with the encapsulated active [47,56]. The choice of lipids used or quality can have considerable impact on the resulting characteristics of the formulation, such as storage stability and particle size [89]. The degree of crystallinity affects the density and packing of the lipid matrix, where highly crystalline structures exhibit reduced molecular mobility and lower diffusion rates, thereby enhancing the retention of volatile EOs but at the cost of reduced incorporation. This makes less ordered structures, like NLCs, more effective for complex oils, where total encapsulation of the oil is the objective as supposed to a singular or substituent group of components [54,55]. Crystallisation behaviour is affected by the production method, melting point of the chosen lipid, lipid concentration, presence/absence of a surfactant, dispersity of particles and particle size [88].

Thermal properties, particularly melting temperature and polymorphic transitions play a central role in determining the stability and release behaviour of SLNs and NLCs. Upon cooling, lipids may transition between different polymorphic forms, with more stable forms exhibiting tighter molecular packing. These transitions can lead to changes in EE and release behaviour over time [89]. Release from SLNs and NLCs is controlled by a combination of diffusion, lipid matrix restructuring and, in some cases, lipid degradation. Diffusion occurs through the lipid matrix, where the rate is influenced by the degree of crystallinity and the localisation of the active compound within the particle. In lipid carriers, compounds located near the particle surface exhibit an initial burst release, whereas those embedded within the core are released more slowly via diffusion through the lipid matrix over time and can be described using Fick’s second law of diffusion [90,91].

#### 3.2.3. Composition and Physicochemical Principles of Polymeric Nanoparticles

PN systems are controlled by principles of self-organisation, intermolecular interactions and mass transport phenomena, where the formation of the encapsulated structure arises from the physicochemical behaviour of polymer chains in solution. Unlike vesicular systems, which rely on amphiphilic bilayer formation, polymeric systems are typically based on the formation of a three-dimensional network or matrix [92]. Encapsulation may occur through several mechanisms depending on the system design, including matrix-type entrapment, core-shell formation or nanoparticle self-assembly. In matrix systems, the active compound is dispersed throughout a continuous polymer phase, whereas in core-shell structures, the polymer forms a distinct barrier surrounding the encapsulated material [61]. These structural differences influence diffusion behaviour, release kinetics and protective capabilities. Polymers often used in EO encapsulation include natural biopolymers such as polysaccharides (e.g., chitosan) [61], proteins (e.g., gelatine) [93] and synthetic polymers (e.g., PLGA) [62]. Encapsulation is stabilised through a combination of electrostatic interactions between positively charged chitosan polymers and negatively charged liposomes, which promote the formation of a stable complex alongside hydrogen bonding and hydrophobic interactions between the polymer matrix, phospholipid bilayer and compatible EO components [61]. The strength of these interactions depends on the polarity, molecular weight and functional groups of both the polymer and the encapsulated compounds, which is discussed in Section 6 [94].

Particle size and morphology are critical determinants of stability, release kinetics and bioavailability in polymeric systems. They may range from microcapsules (>1 µm) to nanoparticles (<1000 nm), depending on the preparation method, and they may exist as spherical nanoparticles, porous matrices or irregular aggregates [80]. The shape and arrangement of the molecules, i.e., dense vs. porous, strongly influences diffusion pathways and therefore release rates [61,62].

Thermal properties and the stability of polymeric systems is described by parameters such as glass transition temperature (Tg) and melting temperature. Below Tg, polymers exist in a rigid, glassy state, with limited molecular mobility. This leads to reduced diffusion and enhanced retention of encapsulated compounds. Above Tg, increased chain mobility facilitates diffusion and release [95,96]. Release from polymeric encapsulation systems is governed by mass transport processes, which differ fundamentally from vesicular systems. Rather than diffusion across a lipid bilayer, release occurs through Fickian diffusion through the matrix [62], polymer swelling [80,95] and relaxation or matrix erosion/degradation [97]. Fick’s second law is often used to describe diffusion-controlled release or simple diffusion. With EOs, smaller, more volatile molecules diffuse more rapidly, while stronger polymer-compound interactions can result in delayed release [90,98]. Swelling, on the other hand, describes the process by which polymeric matrices adsorb a solvent, typically water, leading to an increase in volume and structural relaxation of the polymer network [80]. This phenomenon is driven by osmotic pressure gradients and polymer-solvent interactions, where the penetration of water molecules reduces intermolecular forces between polymer chains and increases chain mobility [95]. As solvent uptake progresses, the polymer transitions from a glassy or semi-rigid state to a more rubbery, hydrated state, resulting in the expansion of pore size and increased free volume within the matrix. This in turn enhances the mobility of encapsulated compounds, thus facilitating their release [80,95]. Highly crosslinked polymers tend to display limited swelling due to restricted chain mobility, whereas loosely crosslinked systems absorb greater quantities of water and swell quicker. Excessive swelling may also compromise the structural integrity, leading to bursting or premature release [95]. Matrix erosion or degradation represents a third major mechanism of release, whereby the polymer structure itself is progressively broken down, facilitating release. This process may occur through chemical degradation, e.g., hydrolysis, enzymatic activity or physical disintegration, depending on the nature of the polymer and surrounding environment [80,97]. In degradable systems, the integrity of the polymer matrix diminishes over time, resulting in increased porosity, reduced release barriers and even structural collapse [97].

#### 3.2.4. Composition and Physicochemical Principles of Inclusion Complexes

The physicochemical behaviour of ICs is managed by principles of host–guest chemistry, molecular recognition, thermodynamic equilibrium and molecular interactions rather than the formation of discrete particulate structures [99]. In these systems, stability arises from reversible, non-covalent association between the guest molecule and the CD cavity, resulting in a dynamic equilibrium between free and complexed species in solution [99,100]. Consequently, key functional attributes such as complexation efficiency binding constant, EE, solubility enhancement, molecular stoichiometry, thermal stability and release characteristics are also dictated by the nature of these interactions rather than by physical confinement [99,100]. Molecular size, shape and steric complementarity of the EO to be encapsulated therefore plays a critical role. The CD cavity is relatively rigid, and optimal binding occurs when the guest molecule achieves minimal surface contact with the internal cavity walls [101]. This “goodness-of-fit” enhances van der Waals interactions and minimises void space within the cavity, which in turn enhances overall stability. Molecules that are too large cannot be accommodated, whereas those that are too small may not form sufficiently stable complexes due to limited contact with the cavity walls. Optimal binding occurs when the guest molecule achieves a close geometric fit to the CD [102]. In EO systems, monoterpenes and phenolic compounds often exhibit favourable compatibility with β-CDs, adopting orientations in which hydrophobic moieties are inserted into the cavity while polar functional groups remain near the rim. This orientation influences both complex stability and the degree of protection against oxidation and volatilisation [49,99]. The driving forces of complexation are thermodynamic in nature and arise from a combination of van der Waals interactions, hydrogen bonding, hydrophobic effects and desolvation phenomena. Among these, van der Waals forces, i.e., dispersion, are particularly significant in well-fitting systems, where close interaction between host and guest maximises interaction energy. Hydrogen bonding may occur at the CD rim, contributing to stabilisation and orientation of the guest molecule [100].

Environmental factors such as pH, temperature and solvent composition significantly influence IC stability. Ionisation of the guest molecule generally reduces affinity for the hydrophobic cavity, while temperature can both enhance solubility and weaken intermolecular interactions. Solvent molecules capable of entering the CD cavity may compete with the guest, reducing binding efficiency. In multicomponent systems such as EOs, selective complexation can occur, where certain constituents exhibit higher affinity, leading to preferential encapsulation and altered compositional profiles [49]. The physicochemical consequences of complexation are substantial. Encapsulation within the CD cavity reduces the exposure of guest molecules to external stressors such as oxygen, light and heat, thereby enhancing chemical stability and reducing degradation. Volatile compounds exhibit decreased vapour pressure and reduced evaporation rates, while poorly soluble compounds display increased apparent aqueous solubility due to the hydrophilic exterior of the complex [49,100]. Release behaviour is governed by equilibrium-driven dissociation rather than diffusion through a physical barrier. As the concentration of free guest in the surrounding environment decreases, the equilibrium shifts towards dissociation, resulting in rapid release of the encapsulated compound. This reversible mechanism distinguishes ICs from vesicular and polymeric systems [100].

## 4. Preparation Methods

### 4.1. Preparation Techniques for Emulsions

**Figure 2:**
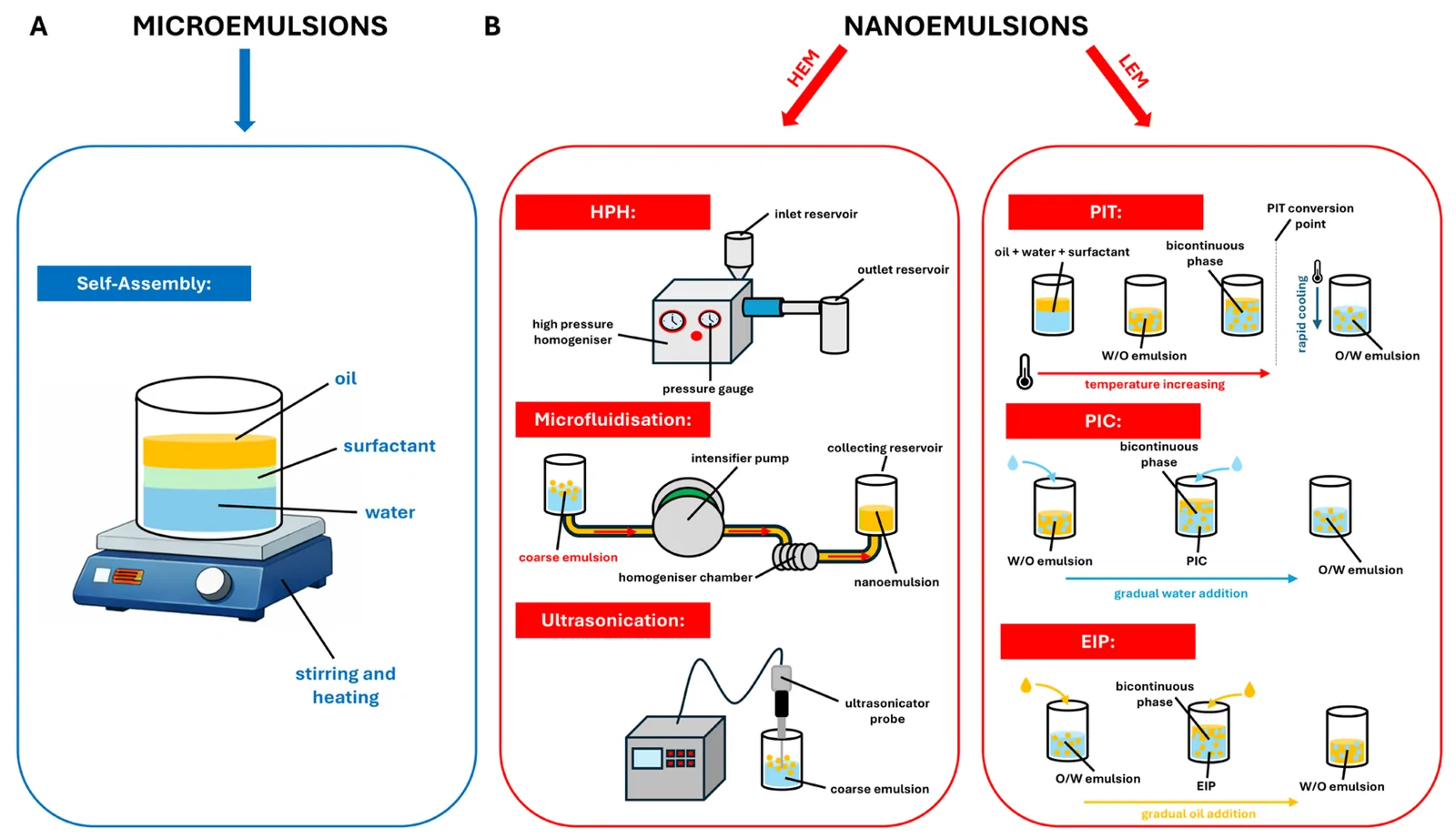
Emulsification preparation methods for (**A**) microemulsions and (**B**) nanoemulsions. Water phases are depicted in blue and oil phases in yellow.

In principle, microemulsions require only oil, water and surfactant to be brought together to be formed spontaneously [39]. However, self-emulsification processes have proved ineffective for EOs, and in order to produce uniform droplets and stable emulsions, external force is required [76]. Thus, stirring or heating is often applied to facilitate the process due to the presence of some kinetic energy or mass transport barriers (Figure 2A).

Opposingly, nanoemulsions require a greater input of external energy [39]. It must exceed the positive free energy associated with increasing the contact area between the oil and water phases. Two methods are thus employed to reach this state, high-energy methods (HEMs) or low-energy methods (LEMs). HEMs utilise mechanical devices to disrupt coarse emulsions into nanoscale droplets. High-pressure homogenisation (HPH) [103], micro fluidisation [104] and ultrasonication [105] are commonly applied techniques (Figure 2B). The droplet size decreases with increasing pressure, number of passes or sonication intensity. LEMs, on the other hand, rely on the spontaneous formation of nanoscale droplets through physicochemical changes in the system rather than intense mechanical forces. Common approaches include spontaneous emulsification, phase inversion temperature (PIT), phase inversion composition (PIC) and the emulsion inversion point (EIP) method (Figure 2B). In spontaneous emulsification, a surfactant–oil mixture is added to an aqueous phase under gentle stirring, causing the formation of fine droplets. In PIT, the emulsion is heated to the PIT and then rapidly cooled while stirring, leading to droplet formation during the inversion process [39]. In PIC and EIP methods, nanoemulsions are formed by gradually changing the composition of the system by, for example, adding water or oil, until phase inversion occurs [106,107]. Droplet formation in LEMs is driven by changes in interfacial curvature and surfactant affinity, allowing nano-sized droplets to form with relatively low energy input. HEMs offer greater versatility in oil and surfactant selection overall, whereas LEMs often require higher surfactant concentrations and specific system compositions. Approaches such as spontaneous emulsification and phase inversion methods may produce either nanoemulsions or microemulsions depending on formulation parameters, especially the surfactant-to-oil ratio [39].

### 4.2. Preparation Techniques for Encapsulated Systems

#### 4.2.1. Approaches for Vesicular Systems

**Figure 3:**
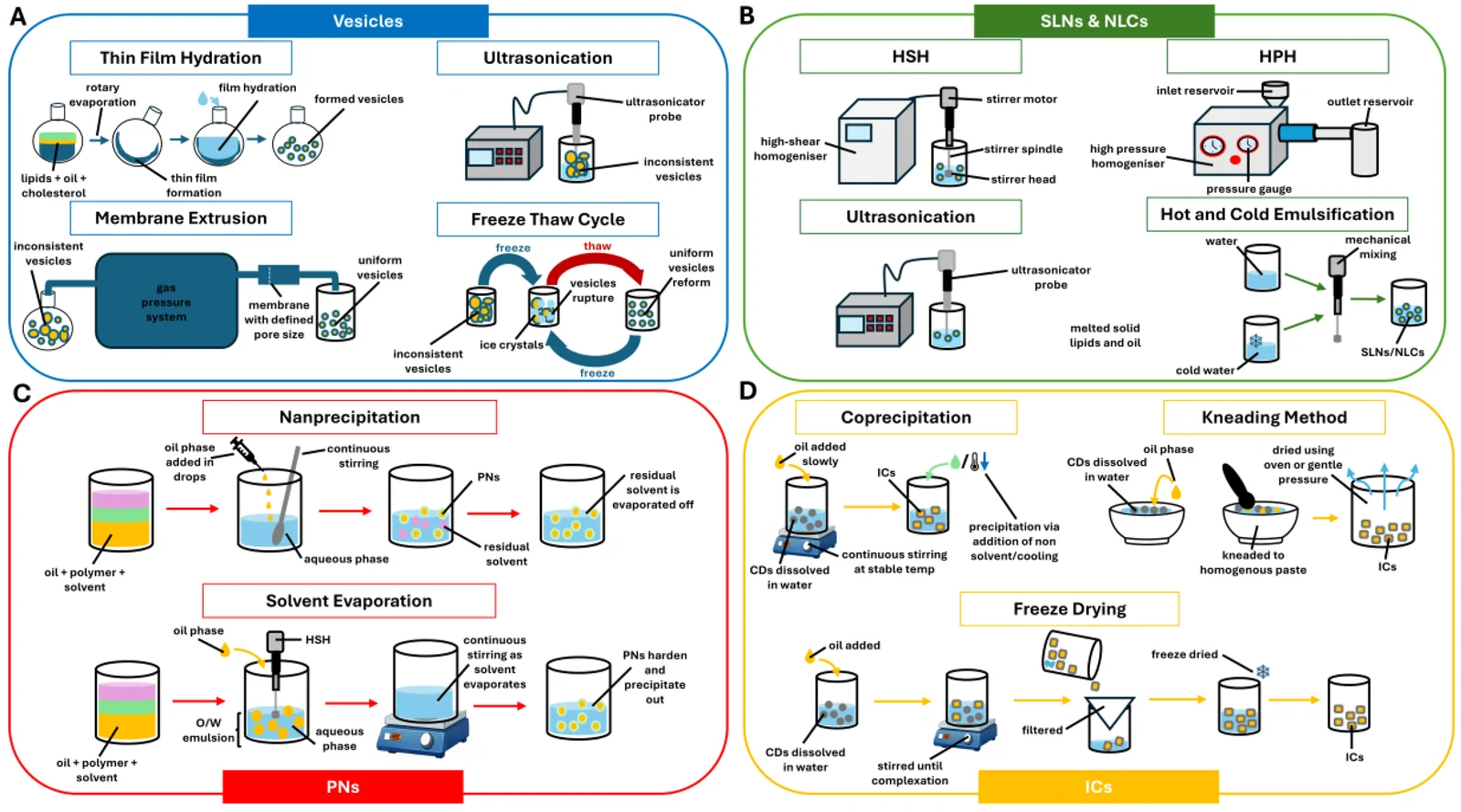
Encapsulation preparation methods for (**A**) vesicles, (**B**) SLNs and NLCs, (**C**) PNs and (**D**) ICs.

EOs, due to their lipophilic nature, are typically incorporated into the lipid bilayer during the vesicle formation stage. Common methods for the formation of liposomes include mechanical dispersion (e.g., thin-film hydration, sonication, membrane extrusion and freeze–thaw) and solvent dispersion (e.g., reverse-phase evaporation and ethanol injection) (Figure 3A) [48,108,109].

Thin-film hydration is the most widely used technique for liposome preparation [110]. Lipids are dissolved in an organic solvent, typically chloroform, and evaporated under reduced pressure to form a thin lipid film. Upon hydration with an aqueous phase, the film swells and detaches, forming MLVs. EOs are typically co-dissolved with the lipid phase prior to evaporation to facilitate incorporation into the bilayer [48,108]. Factors such as hydration time, temperature and lipid-to-oil ratio significantly affect EE and vesicle size. This method has been successfully applied in numerous studies encapsulating EOs [111,112,113]. However, due to the volatile nature of EO constituents like monoterpenes, some of the EO may be lost during the evaporation step. In contrast, for oils like clove and cinnamon, whose primary constituents are phenylpropanoids and sesquiterpenes [114,115], the lower volatility of these compounds reduces evaporative losses, making them more suitable for such techniques. The thin-film hydration method is simple and versatile but generally produces heterogenous, large vesicles that require further size reduction via extrusion and sonication [48]. Sonication is suitable for heat- or shear-resistant EOs; however, it may produce liposomes with low EE, with possible degradation of the phospholipids and compounds encapsulated [116]. Extrusion provides uniform size distribution and a narrow PDI, with often larger SUVs than if sonicated, as demonstrated by Arabi et al. [111], who showed that liposomes containing rosemary EO were greater in size (470 nm) when extruded versus sonicated (162 nm).

Reverse-phase evaporation, a solvent dispersion method, yields SUVs with high encapsulation of hydrophobic compounds. Lipids and EOs dissolved in organic solvents are emulsified with aqueous media. Removal of the organic phase under reduced pressure leads to vesicle formation [109]. The technique is particularly effective for EOs prone to leakage during conventional hydration and has been applied to several EOs for the enhancement of their antimicrobial efficacy [117]. However, the main disadvantage of this technique is the exposure of the encapsulated materials to brief periods of sonication during size reduction steps and to organic solvents [108,118]. In contrast, the injection method (ethanol/ether) eliminates the need for an additional size reduction step [118]. Control over injection rate, lipid concentration, and temperature allows for the formation of SUVs suitable for scalable production. This method has been successfully applied to clove EO by Sebaaly et al. [86], where stable, spherical liposomes were produced and protected eugenol from UV-mediated exposure.

Freeze–thaw cycle and supercritical fluid methods are less common in EO vesicle encapsulation; however, there are some publications that reference these as either the primary encapsulation technique or as a potential avenue for method optimisation [119,120]. The freeze–thaw technique is typically employed as a post-processing step following conventional liposome formation, such as thin-film hydration [119,121]. Supercritical fluid methods enable solvent-free or reduced processing, which is advantageous for EOs. This process can yield liposomes with narrow size distributions and improved EE while minimising solvent contamination [120,122]. Despite these benefits, the requirement for specialised equipment and the complexity of process optimisation have limited their widespread adoption in EO delivery systems [122]. For the same reason and potentiality for high toxicity, detergent solvent methods are underexplored in EO research even with them being established in liposome technology [123]. Among the available preparation techniques, thin-film hydration and ethanol injection dominate in EO encapsulation studies, owing to their simplicity and compatibility with lipophilic compounds.

#### 4.2.2. Approaches for Lipid Carriers

High-Shear Homogenisation (HSH) is one of the most broadly used techniques for the preparation of SLNs and NLCs (Figure 3B). In this method, the lipid phase containing the dissolved active is melted above its melting point and dispersed into a hot aqueous phase containing surfactants under high shear mixing. The resulting coarse emulsion is then cooled, leading to lipid recrystallisation and nanoparticle formation [89]. HSH offers advantages in terms of simplicity, scalability and avoidance of organic solvents, making it exceptionally attractive for food and pharmaceutical applications. However, this method often produces larger particle sizes with broad size distributions compared to other methods, necessitating further size reduction [124,125]. Additionally, the elevated temperatures required may lead to the degradation or volatilisation of sensitive components [53]. HSH was efficiently applied to clove EO to form SLNs that were successful in antimicrobial assays against Gram-positive and Gram-negative and some fungal species. Increased levels of surfactant improved the size of the SLNs and therefore the stability; however, maximum encapsulation is reported to be around only 70%, meaning that some EO is lost during preparation [126].

High Pressure Homogenisation (HPH) is considered the gold standard for SLN and NLC production and can be performed using either hot or cold homogenisation techniques (Figure 3B) [89]. In hot homogenisation, the lipid phase containing the EO is melted and emulsified in a heated aqueous surfactant solution, followed by passage through a high-pressure homogeniser. The resulting nanoemulsion is then cooled, leading to the formation of SLNs [89]. This method allows for the production of particles with relatively small sizes and narrow size population distributions while being highly scalable for industrial applications. However, similar to HSH, the use of elevated temperatures may result in the partial loss or degradation of volatile EO components as well as potential partitioning of the EO into the aqueous phase during homogenisation [55]. Cold homogenisation was developed to address these limitations. In this approach, the lipid phase containing the EO is rapidly cooled and solidified, typically using liquid nitrogen or dry ice, and then milled into microparticles. These solid lipid particles are subsequently dispersed in a cold aqueous surfactant solution and subjected to HPH at or below room temperature [89]. This method minimises the thermal degradation and volatilisation of EO constituents and reduces their diffusion into the aqueous phase. Nonetheless, it often results in larger particle sizes and broader size distributions compared to hot homogenisation and may exhibit lower EE due to incomplete incorporation of the active compound [89,125]. Both hot and cold HPH methods have been effectively utilised in the encapsulation of EOs in both SLNs and NLCs [57,127] for boosted antimicrobial activity and elongated storage stability. Reports demonstrate a good EE when the method is optimised, albeit a varied population distribution, particle size [57] and only short-term stability (~12 days) [127].

Hot and cold emulsification techniques are closely related to homogenisation-based approaches and are frequently employed in SLN and NLC preparation (Figure 3B). In hot emulsification, similar to hot homogenisation, the lipid and aqueous phases are combined at elevated temperatures, forming an emulsion that solidifies upon cooling. In contrast, cold emulsification involves the dispersion of SLNs into a cold aqueous phase, followed by mechanical size reduction. These techniques provide flexibility in tailoring nanoparticle characteristics; however, EO studies that have employed them report poor EEs (<40%) and large particle sizes (>600 nm) [128].

Among the available techniques, HPH is the dominant method for the preparation of EO-loaded lipid carriers, producing stable formulations for lavender, oregano, rosemary [58], clove [126] and Shirazi thyme EOs [59,129,130]. HPH remains the most widely applied method due to its scalability and ability to produce uniform particles, while cold homogenisation offers an alternative for thermolabile actives. In contrast, solvent-based and microemulsion-derived approaches are less frequently used for EO formulation, while methods such as phase inversion or membrane-based techniques remain largely unexplored.

#### 4.2.3. Approaches for Polymeric Nanoparticles

The most widely employed preparation methods for PNs with EOs include nanoprecipitation and solvent evaporation, each offering distinct advantages and limitations, depending on the intended application (Figure 3C) [131,132,133]. Ionic gelation, albeit a widely used technique, requires pre-emulsification of the EOs before being encapsulated, as the method is geared towards fully water-soluble systems [132].

Nanoprecipitation is another commonly employed technique for the preparation of PNs [134]. In this method, the polymer and oil are dissolved in a water-miscible organic solvent such as acetone or ethanol and added to an aqueous phase containing a stabiliser under controlled mixing conditions [134]. Addition of the acetonic-oily solution results in spontaneous emulsification. Rapid diffusion of the organic solvent into the aqueous phase leads to supersaturation of the polymer and the spontaneous formation of nanoparticles [134]. This technique is particularly advantageous for producing small particles with narrow size distributions and relatively low PDI, without the need for high energy input. It is also regarded widely as a simple, rapid and reproducible method, with nanoparticle formation occurring almost instantaneously following solvent displacement [131]. For EO encapsulation, nanoprecipitation can achieve good loading capacity for hydrophobic constituents due to their affinity for the polymer matrix. However, the use of organic solvents introduces concerns regarding residual solvent toxicity and potential loss of volatiles during solvent diffusion and evaporation. Furthermore, the method is generally limited to polymers that are soluble in water-miscible solvents, restricting formulation flexibility [135]. Several studies have examined how to optimise this method for EO encapsulation [136,137]. Liakos et al. [136] found that the EO itself, where *Cinnamomum verum* J. Presl. (cinnamon), peppermint and lemongrass were sampled, affected the resulting nanoparticle size. This was attributed to the chemical structure of the molecules present in each EO. Compounds with long carbon chains demonstrated increased particle size. The surface charge of each plant metabolite meant that the overall zeta potential for the formulated nanoparticles was also EO-dependent [136,138]. The addition of surfactant was useful in stabilising rosemary EO-loaded nanoparticles [137]. However, EOs themselves, especially those that contain molecules with long hydrocarbon tails and heads with functional groups as well as some aldehydes, which can create aldol reactions with polylactic esters, can act as stabilisers [139]. As well as the consideration of the type of EO encapsulated, temperature and solvent elimination approach also greatly impact the final particles and EE. Lower temperatures (30 °C) created larger particles than higher temperatures (90 °C) [139]. Evaporation of the organic solvent under reduced pressure at room temperature and a higher temperature (40 °C) saw a 50% loss of EO during the preparation of nanoparticles. Only evaporation of the solvent at normal pressure and room temperature saw a negligible loss of the EO due to the avoidance of heat and low pressure [137].

With regards to the solvent evaporation technique, the polymer and active component are dissolved in a volatile, water-immiscible organic solvent such as dichloromethane, chloroform or ethyl acetate to form the dispersed phase [131,133]. This phase is then emulsified into an aqueous phase containing surfactants or stabilisers using HSH or sonication, producing an O/W emulsion. Subsequent evaporation of the organic solvent by continuous stirring, reduced pressure or filtration leads to polymer precipitation and nanoparticle formation [131]. This method allows for the high EE of lipophilic EO components and offers good control over particle size through the adjustment of emulsification parameters. It is also widely adaptable to different polymers, including biodegradable materials like PLGA. Despite its well-established advantages, its use in the formulation of EO-loaded nanoparticles remains relatively limited [140], largely due to challenges associated with the volatility and physicochemical instability of these compounds under processing conditions. The requirement for high energy input during emulsification may lead to the degradation of sensitive EO components, while the use of organic solvents raises safety, environmental and regulatory concerns [131]. Lastly, with this method, there is the potential for burst release, where a fraction of the encapsulated active rapidly diffuses out of the nanoparticles due to surface-associated compounds [133]. Vrouvaki et al. [140] developed PNs encapsulating *Pistacia lentiscus* var. *chia* Poir. (mastic) EO using a solvent-evaporation method for the treatment of minor skin inflammation. The choice of surfactant greatly impacted the EE, release profile, long-term stability, presence of humidity and final antimicrobial activity, with PVA-stabilised PLA nanoparticles achieving notably higher EE lecithin-stabilised counterparts (37.45% vs. 9.15%, respectively). Nevertheless, even with optimisation of the method, encapsulation of the total oil was not reached, with a maximum of 50% observed across all samples.

#### 4.2.4. Approaches for Inclusion Complexes

Among the most widely used techniques for ICs with EOs are co-precipitation, kneading and freeze-drying, each offering distinct advantages depending on the physicochemical properties of the EO components and the intended application (Figure 3D) [49,100].

Co-precipitation is one of the most commonly employed methods for inclusion complexation. In this approach, CDs are dissolved in water to form a saturated or near-saturated solution, followed by the gradual addition of the EO under continuous stirring. The system is maintained under controlled temperature and agitation to facilitate host–guest interaction and IC formation. Following this, cooling or addition of a non-solvent can induce precipitation of the complex, which is then recovered by filtration and drying, centrifugation or decanting [49,99]. This method is relatively simple and suitable for scale up and has been broadly used for encapsulating EOs like *Citrus* × *bergamia* Risso & Poit (bergamot), rosemary and clove [141,142]. In this method, although effective at entrapping hydrophobic molecules, the organic solvents used as the precipitant in non-phase solubility methods may inhibit inclusion, whereas low yield and high water usage in phase solubility-based approaches limit co-precipitation as a technique for EOs [49,99]. Furthermore, Stancu et al. [141] found that whilst co-precipitation-lyophilisation was better at encapsulating terpenes like eucalyptol, the kneading method proved more effective for phenylpropanoids like eugenol.

The kneading method offers a more concentrated and solvent-minimised approach to complex formation. In this technique, CDs are mixed with a small amount of water or hydroalcoholic solution to form a paste, into which the EO is incorporated under mechanical agitation. The mixture is kneaded for a defined period to promote diffusion of the guest molecules into the CD cavity, followed by drying and milling to obtain a solid complex [49,100]. The kneading method is beneficial in terms of reduced solvent usage, simplicity and high complexation efficiency. It is notably suitable for thermosensitive actives, as it avoids high temperatures and extensive processing steps. However, drawbacks of the technique include potential heterogeneity in the final product, dependence on operator-controlled variables such as kneading intensity and duration, and later, uniformity challenges if the production is scaled up [49]. It has been utilised in the formulation of *Chamaemelum nobile* (L.) All. (chamomile) EO CDs [143], demonstrating that increasing the ratio of β-CDs to oil leads to a better total encapsulation. Additional studies have shown that *Psidium guajava* L. (guava), clove, *Callistemon viminalis* (Sol. ex Gaertn.) Byrnes (weeping bottlebrush), *Lippia gracilis* Phil. (lemon verbena) and *Cymbopogon martini* var. *sofia* B.K. Gupta (gingergrass) are among the many EOs to have been encapsulated in ICs under kneading conditions [144,145,146,147].

Drying-based methods, such as freeze-drying, i.e., lyophilisation, and spray-drying are frequently applied to produce stable powdered ICs with better handling and storage properties. In freeze-drying, an aqueous solution containing the CD and target active is first frozen and then subjected to sublimation under reduced pressure, resulting in a porous, amorphous powder [100,148]. This method is particularly effective in minimising thermal degradation, making it highly suitable for EO encapsulation. It also tends to yield high EEs due to the rapid immobilisation of the guest molecules within the CD matrix. However, freeze-drying is energy intensive, time-consuming, and may present scalability challenges, and due to the initial step of mixing water, it is not entirely suitable for hydrophobic guests [49]. Nonetheless, a study by Enciso-Sáenz et al. [149] found success in encapsulating lemongrass EO using the freeze-dry method. The authors found that the steric nature of the volatile components impacted the retention efficiency in the CDs, but overall, it was an adequate technique. Spray drying, by contrast, is non-viable for EOs, as they contain thermolabile components, which would degrade during processing due to the elevated temperatures (50–70 °C) required [49].

## 5. Stability Concerns

### 5.1. Considerations for Emulsion Stability

Microemulsions are thermodynamically stable under defined compositional and temperature conditions and do not undergo classical instability phenomena such as coalescence or Ostwald ripening. Only if there is a deviation from the range of environmental conditions where the system is thermodynamically stable might it become unstable [37]. In contrast, the long-term stability of nanoemulsions depends on the height of any energy barriers between the emulsified state and the separated states and its resistance to destabilisation mechanisms such as creaming, flocculation, coalescence and Ostwald ripening [4,37]. Oils rich in relatively water-soluble monoterpenes, e.g., thyme EO containing thymol, carvacrol, eugenol and linalool, exhibit greater molecular diffusion through the continuous phase, promoting droplet growth and eventual destabilisation [41]. Conversely, EOs containing larger proportions of higher-molecular-weight or oxygenated constituents, such as cedrol in *Cedrus* sp. (cedarwood) EOs [150] and caryophyllene and patchoulol in *Pogostemon cablin* Benth. (patchouli) EO [151], will generally display lower rates of Ostwald ripening owing to their reduced aqueous solubility. This will draw into question their storage stability over time. By incorporating stabilisers such as emulsifiers, ripening retarders, weighting agents, and texture modifiers, long-term stability can be improved [37]. Notably, many published studies report only short-term stability for EO-based nanoemulsions (30–60 days) [43,152,153].

### 5.2. Considerations for Encapsulated System Stability

#### 5.2.1. Vesicular Systems

Vesicular systems are generally considered kinetically stable, and their long-term stability is determined by both physical and chemical instability events. However, as they are not thermodynamically stable like microemulsions, they tend to undergo gradual changes over time, like vesicle aggregation, fusion and leakage of encapsulated compounds [154,155]. Physical instability is often manifested through increases in vesicle size and PDI due to fusion or flocculation [156,157], while chemical instability primarily involves oxidation or hydrolysis of phospholipids, particularly in the presence of unsaturated lipid components [157,158]. These processes can significantly impact the retention of EOs within the lipid bilayer. Additionally, in EO-loaded liposomes, stability challenges are further compounded by the volatile and chemically labile nature of the metabolites. In addition, due to their lipophilic nature, EOs are primarily localised within the lipid bilayer, where they can disrupt membrane packing and increase bilayer fluidity; this may enhance permeability and result in premature leakage of the EO during storage [48]. Consequently, careful consideration of the lipid composition, including incorporation of cholesterol and/or saturated phospholipids, is often required for EO-loaded vesicles [82]. Strategies such as the inclusion of antioxidants to limit oxidation, surface modification using polymer coatings to reduce aggregation, and lyophilisation with appropriate cryoprotectants to enhance the long-term storage stability should be considered [155,157]. Instead, only short- to medium-term stability data exist [159].

#### 5.2.2. Lipid Carriers

SLNs and NLCs are generally considered physically stable systems due to the presence of a solid lipid matrix, which provides greater structural rigidity compared to emulsions or vesicular systems. However, SLNs, which are typically used more for EOs [126,127,128], are especially prone to polymorphic transitions during storage, whereby the lipid matrix transforms from a less ordered system into a more stable crystalline structure. This transition can result in increased lipid packing density and the subsequent expulsion of encapsulated EO compounds [47,53,89,160]. This phenomenon may be particularly relevant for citrus EOs, rich in limonene, where the loss of volatile monoterpenes following lipid rearrangement can reduce both aroma retention and biological activity [161]. NLCs, although developed to address this limitation, have been utilised rarely in EO research [57], as although NLCs offer theoretical advantages over SLNs in terms of loading capacity and reduced expulsion, formulation complexity and additional preparation steps remain inherent limitations of this approach.

Physical instability in SLNs and NLCs typically manifests through particle aggregation, gelation or increases in particle size and PDI, often due to insufficient steric or electrostatic stabilisation [89]. Lipid crystallisation and recrystallisation can further alter particle morphology over time, affecting EE and release behaviour. Chemical instability is primarily driven by lipid oxidation, especially in systems containing unsaturated lipids or oxidation-prone EO components, leading to degradation products that compromise both nanoparticle integrity and cargo stability [53,55]. This is particularly important for EOs rich in readily oxidisable constituents, such as clove (eugenol-rich) and thyme oil (thymol-rich), where oxidation may occur despite encapsulation if oxygen permeates the lipid matrix during storage [161]. Studies involving eucalyptus and rosemary EO-loaded SLNs and NLCs have demonstrated that EO incorporation can influence particle characteristics and long-term stability [162]. Additionally, EOs can interact with the lipid matrix, disrupting crystallisation behaviour and acting as plasticisers, which may either enhance loading or destabilise the system by increasing matrix permeability and promoting premature release [128]. To improve stability, formulation strategies include selecting appropriate lipid compositions, such as incorporating liquid (unsaturated) lipids into the solid matrix of NLCs to disrupt crystallisation and reduce the expulsion of encapsulated compounds, and using surfactants to maintain colloidal stability [55,89]. Souza et al. [163] demonstrated that oregano-containing NLCs showed degradation in their overall quality when they surpassed a heating threshold during preparation.

#### 5.2.3. Polymeric Nanoparticles

While these systems are often considered kinetically stable, they are not immune to instability. Physical instability may manifest as particle aggregation, sedimentation or changes in particle distribution and PDI, particularly in systems lacking adequate steric or electrostatic stabilisation, and it can be measured by monitoring pH as a function of time [135]. Chemical instability in PNs is largely associated with polymer degradation, typically via hydrolysis and, to a lesser extent, oxidative processes. Biodegradable polymers such as PLGA undergo hydrolytic cleavage of ester bonds, leading to gradual erosion of the polymer matrix and the release of encapsulated compounds [132]. While this behaviour is often desirable for controlled release applications, it may result in premature leakage or unpredictable release profiles under certain storage or environmental conditions [132]. Additionally, the interaction between EO and the polymer matrix may influence nanoparticle stability. Lipophilic compounds may act as plasticisers within the polymer, altering its mechanical properties, increasing matrix permeability and potentially accelerating diffusion-driven release [139]. For example, studies involving PLGA nanoparticles loaded with carvacrol and p-cymene have demonstrated that differences in non-covalent interactions between encapsulated compounds and the polymer matrix affect release kinetics and encapsulation behaviour [164]. Carvacrol contains a phenolic hydroxyl group capable of hydrogen bonding with the ester carbonyl groups of PLGA, increasing its affinity for the polymer matrix and slowing diffusion. Conversely, p-cymene lacks polar functional groups and interacts predominantly through weaker hydrophobic and van der Waals interactions, resulting in reduced matrix affinity and more rapid diffusion. Thymol and p-cymene have been shown to exhibit distinct release profiles from polymeric matrices, highlighting the importance of EO composition when designing stable PN formulations [165]. Comprehensive long-term stability data remain limited.

#### 5.2.4. Inclusion Complexes

The stability of ICs relies on molecular size compatibility between guest and cavity, polarity and functional groups of the EO compound and their association, and the temperature and solvent environment [148]. This has been demonstrated for citrus EOs, where limonene is the dominant constituent, and for thyme and oregano EOs, which contain high concentrations of thymol and carvacrol. These compounds exhibit molecular dimensions that are highly compatible with CD cavities [166,167,168,169]. In contrast, EOs rich in sesquiterpenes, such as clove and *Piper nigrum* L. (black pepper), may display lower EEs due to steric limitations associated with larger molecular structures such as β-caryophyllene [99,148,170,171]. Physical instability is associated with moisture uptake, crystallinity changes and phase transitions during storage [172]. ICs may exist in either crystalline or amorphous states, with amorphous forms generally exhibiting higher apparent solubility but lower physical stability due to a tendency towards recrystallisation over time [49]. As well as this, competition between water molecules and guest compounds for occupancy within the CD cavity promotes displacement and release of the guest molecule or molecules in the case of EOs, which are already competing with each other for inclusion. This is notably relevant under high humidity storage conditions, where partial decomplexation and loss of volatile compounds may occur [49,100,148,172]. Chemical instability, on the other hand, is primarily connected to the intrinsic reactivity of the encapsulated components rather than the degradation of the IC itself. Although encapsulation reduces exposure to environmental stressors, EO constituents may still undergo oxidation, isomerisation or hydrolysis if partially exposed or released from the cavity [8,49,99]. Furthermore, the equilibrium nature of inclusion means that a fraction of the EO molecules remains unincorporated at any given time, rendering them susceptible to degradation. The extent of protection is therefore highly dependent on the association and complexation efficiency. Weakly bound complexes may provide limited stabilisation, particularly for highly volatile and bulky monoterpenes, e.g., eucalyptol [173]. From a compositional standpoint, the stability of ICs is strongly influenced by the type of CD used. Native β-CDs exhibit a relatively low aqueous solubility, which may limit their effectiveness in certain applications, whereas modified CDs such as HP-β-CDs or methylated derivatives offer improved solubility and complexation efficiency but may differ in terms of stability and safety profiles [148].

## 6. Essential Oils in Formulation

### 6.1. Current Research

#### 6.1.1. Current Essential Oil-Based Emulsion Studies

Emulsification represents one of the most widely investigated and practiced strategies for the incorporation of EOs into functional delivery systems, owing to their relative simplicity, scalability, compatibility with aqueous matrices and ability to incorporate the total oil profile with minimal selectivity for certain compounds. The most influential aspect of an EO emulsion is the plant from which it is derived, which strongly affects the surfactant selection, emulsification technique and long-term stability. In terms of surfactants, polysorbates (Tween series) are consistently reported as highly effective surfactants across a wide range of EOs, regardless of their chemical composition [76,174]. In contrast, more lipophilic surfactants like Span tend to be more selective towards the composition of the oil phase in the emulsion. For example, *Pimpinella anisum* L. (anise) and *Foeniculum vulgare* Mill. (fennel) EOs, which are primarily made up of phenylpropanoids, generate a dense foam in the presence of Span 80 that hinders the application of further sonication steps. Campolo et al. [76] attributes this foaming event to the presence of (*E*)-anethole, a significant compound in both fennel and anise EOs. The compositional effects of an EO on the emulsification preparation steps are further highlighted by Salvia-Trujillo et al. [175], who showed that in a range of EOs formulated for food flavourings and preservatives, *Cymbopogon martini* (Roxb.) Will. Watson (palmarosa) and *Aniba rosodora* Ducke (rosewood) EOs reached the desired droplet size following a sole HPH step, whereas the other EOs in the study required an additional microfluidisation step for nanoscale reduction. Sensory characteristics such as appearance also varied between the oils, with *Melaleuca alternifolia* (Maiden & Betche) Cheel (tea tree), *Origanum majorana* L. (marjoram) and *Pelargonium graveolens* L’Hér (geranium) EO nanoemulsions being completely transparent as supposed to slightly creamy like the others, indicating stability implications dependent on the EO used. It is evident from several studies that the size and surface charge of droplets is also reliant on the type of EO. Campolo et al. [76] demonstrated that *Artemisia vulgaris* L. (artemisia) EO was the most successful emulsion in terms of droplet size stability out of a range of seven EOs. This is largely due to its composition being primarily oxygenated monoterpenes (~80%). On the other hand, the most unstable EO, *Salvia officinalis* L. (sage), had a more diversified profile, with varying percentage concentrations of monoterpene hydrocarbons, oxygenated monoterpenes and sesquiterpenes. Whilst the complexity of sage EO makes it harder to predict the effects of surfactant and preparation method on the final formulation, it was deduced that the high presence of sesquiterpenes was the cause for reduced stability, as lavender EO, which also presents a high concentration of oxygenated monoterpenes (~90%) and a significant proportion of sesquiterpenes (4.3%), also showed instability comparable to that of sage. Surface charge, another indicator of stability, is also impacted by the composition of the EO, the pH and electrolytes present in the water phase [176]. Campolo et al. [76] illustrated that formulated anise EO had a stable surface charge (~−18 mV), whereas fennel and rosemary displayed fluctuating values over the storage period (−26 to −12 mV) despite formulation preparation being consistent.

With droplet size and surface charge varying dependent on the EO, the observed bioactivity also ranges on the basis of the differences in these physicochemical factors. Clavijo-Romero et al. [177] showed that both the loading rate and the droplet size of the EO within the emulsion affected the observed bioactivity. In *Eucalyptus globulus* Labill. (eucalyptus) EO-based emulsions, emulsions with a higher volume of EO regardless of droplet size or a lower volume with smaller droplet size showed the best bactericidal effects in a death kinetics model for *Escherichia coli, Staphylococcus aureus* and *P. aeruginosa*. The same was true for clove and lemongrass EO nanoemulsions, which showed greater antimicrobial effects against *E. coli* when droplet size was reduced as opposed to the original coarse emulsion. Eugenol-loaded nanoemulsions also showed increased antioxidant and antimicrobial efficacy due to improved dispersion and interfacial interaction with microbial membranes [175].

Collectively, these studies demonstrate that while emulsification is a competent strategy for enhancing the delivery and performance of EOs, especially when total oil incorporation is the objective, the success of the formulation is highly dependent on understanding the oil’s chemical composition. This, in turn, informs the formulation approach to be undertaken.

#### 6.1.2. Current Essential Oil-Based Encapsulation Studies

Among encapsulated systems, liposomes demonstrated the greatest affinity for EO incorporation, with several studies reporting EEs exceeding 90%. For instance, eucalyptus EO achieved around 95% encapsulation [119], while *Anethum graveolens* L. (dill) EO reached 98% [178]. Despite these high values, EE remains highly dependent on the individual constituents of the EO, as demonstrated by the markedly lower incorporation of pure carvacrol (4%) in these vesicles [179] and the variable encapsulation of eugenol (56–84%) as a result of preparation method [86]. Interestingly, reduced encapsulation does not necessarily correlate with reduced functionality, as several studies report enhanced antimicrobial activity even at lower incorporation levels, suggesting that the interfacial localisation of EO components may contribute to bioactivity [86].

The physicochemical properties of liposomes are also strongly influenced by EO loading. Increasing EO concentration typically results in larger vesicle sizes and higher PDIs, indicating reduced system uniformity [86]. This effect has been linked to specific plant metabolites strongly interacting with the lipid bilayer. Components like citral, limonene, eucalyptol and eugenol have demonstrated a level of instability when incorporated at increasing concentrations. With eugenol, the increase in vesicle size is due to the strong interactions with liposomes due to the hydrophobic interaction between its allyl group and the acyl chains of the lipids [86]. A maximum stable loading threshold for an EO has to be identified first; once within this range, stability indicators such as PDI and zeta potential remain comparable to unloaded liposomes [86]. Notably, the choice of lipid appears less influential than EO composition, with Sinico et al. [6] reporting negligible differences in EE between hydrogenated and non-hydrogenated phospholipids for *Artemisia arborescens* L. (wormwood) EO. In contrast, lipid carriers have been less extensively explored for EO delivery; primary research tends to be focused on food packaging systems. Consequently, the criteria used to evaluate their performance are often less stringent. Reported PDI values frequently exceed the threshold for monodispersity (PDI < 0.3), with values of 0.36 for Shirazi thyme EO lipid carriers [59] and 0.4 for peppermint EO lipid carriers [180], indicating broader particle size distributions when compared with vesicles and ICs. Despite this, EEs are typically around 80%, and improvements in biological activity have still been observed [59]. For example, Shirazi thyme formulated in SLNs exhibited enhanced antifungal activity against *Alternaria solani* (77%) compared to the pure oil (54%) [59]. However, this enhancement is not universal. *Cuminum cyminum* L. (cumin) EO encapsulated in NLCs showed lower initial antioxidant activity than the free oil (19–67% vs. 97%, depending on the formulation), though notably, the NLC-formulated EO retained this activity over 30 days of storage, whereas free EO antioxidant activity declined significantly [181]. In the last decade, NLCs have had particular success in incorporating EOs for wound healing applications. For example, once encapsulated in NLCs, peppermint EO provided showed promise as a successful topical treatment for wounds infected with *E. coli, P. aeruginosa, Listeria monocytogenes, Staphylococcus epidermis* and *S. aureus* [180]. Additionally, rosemary and *Mentha pulegium* L. (pennyroyal) EOs were formulated in NLCs for a similar purpose, with success against the same microbial pathogens [182,183]. However, a notable limitation in the EO-based lipid carrier literature is the lack of systematic studies comparing different EOs under identical conditions, generating insight into the individual effects of EO composition on final formulation [129] as well as characterising the EOs used in these studies in general. Therefore, understanding of how the chemical composition affects the reported physicochemical properties or bioactivity is incomplete [180,182,183]. Nonetheless, what can be inferred from these studies is that the EOs used are predominantly composed of volatile, lipophilic monoterpenes and oxygenated monoterpenes (e.g., pulegone and menthone in pennyroyal, menthol, menthone and eucalyptol in peppermint, and eucalyptol and camphor in rosemary). This would suggest that NLCs are well suited to retaining such constituents within their imperfect matrix structure, protecting the EO from volatilisation and prolonged release at the target site.

PNs present a more complex and less predictable system for EO encapsulation, largely due to strong interactions between the EO and polymer matrix. A consistent observation across studies is the swelling of PNs upon EO incorporation, with reports of particle size doubling for bergamot and *Citrus* × *sinensis* (L.) Osbeck (sweet orange) EO-loaded systems [184]. This swelling is accompanied by increased size distribution, indicating reduced homogeneity. Despite successful applications, particularly in agricultural contexts such as insecticidal delivery, PNs often exhibit suboptimal stability, as reflected by wide-ranging zeta potential values that frequently fall outside the range associated with stable dispersions (±30 mV). For example, *Mentha spicata* L. (spearmint) EO formulations show values between −1.01 and −13.6 mV, while peppermint EO exhibits −12.12 mV and *Satureja hortensis* L. (summer savoury) leaves show a range of −7.54 to 21.12 mV [63,185,186]. An additional stability limitation of PNs is the incomplete encapsulation of EO components, with residual oil accumulating on the particle surface. This surface-associated EO forms a “sticky” layer that promotes particle aggregation, a key indicator of formulation instability [185]. Morphologically, this is reflected in the presence of rough, irregular surfaces on otherwise spherical particles, often attributed to both EO localisation and water loss during drying [187]. The selective nature of encapsulation is particularly evident in these systems. Yeguerman et al. [188] demonstrated that peppermint EO-loaded PNs lost several volatile constituents (α-pinene, β-pinene, limonene, eucalyptol, iso-pulegol and pulegone) post-encapsulation, whereas palmarosa EO retained its full compositional profile. These chemical differences translated directly into physicochemical behaviour, with peppermint formulations exhibiting larger particle sizes (381 nm) and high PDI (>0.5) compared to the smaller, more uniform palmarosa particles (191 nm, PDI < 0.25). Palmarosa EO is dominated by geraniol, a relatively alicyclic monoterpene alcohol capable of hydrogen bonding within the polymer matrix, promoting more homogenous incorporation and reduced disruption of nanoparticle formation. In contrast, peppermint comprises a diverse mixture of alcohols, ketones, esters, ethers and terpene hydrocarbons with varying polarity, volatility and molecular structure. Differential partitioning of these compounds likely resulted in both selective encapsulation and varied particle size. Additionally, EE in PNs follows a typical concentration-dependent trend, increasing to a maximum before plateauing and decreasing at higher loadings, although the maximum loading capacity is different for each EO (e.g., sweet orange EO = 6 mg/mL, bergamot EO = 4 mg/mL) [184].

Lastly, stable ICs, particularly those formed with β-CDs [66], are highly dependent on EO composition as well as preparation parameters such as solvent choice and order of addition. For instance, pre-dissolving β-CDs in water prior to EO addition significantly enhances EE [189]. No single solvent has been shown to offer optimal dissolution for all EOs in ICs. Instead, solvent suitability is dependent on the EO itself, e.g., palmarosa EO is more stable in water, whereas *Illicium verum* Hook. f. (star anise) EO shows improved performance in methanol [189]. Independent of solvent choice, it has been shown for palmarosa, garlic and cinnamon leaf EO that the higher the concentration of the oil over a certain point, the higher the EE [189,190]. However, the opposite trend was observed for star anise [189], highlighting the complexity of matching formulation approach with specific EOs. The selectivity of ICs is strongly influenced by the molecular structure of individual EO components. For example, alicyclic molecules such as geraniol exhibit strong encapsulation due to hydrogen bonding interactions. In contrast, (*E*)-anethole interacts with the CD cavity through its methoxy and ethylene groups, forming comparatively strong but structurally different interactions. Larger, more structurally complex molecules such as eucalyptol, a prominent constituent of rosemary EO, experience steric hindrance due to their bulky bicyclic structure, which limits their ability to effectively interact with and fit in the CD cavity. Although not all bicyclic structures, like β-pinene and camphene, show this adversity to complexation, their smaller size ensures there is no problem with cavity fit [66]. These compositional effects are further reflected at the whole-oil level, where clove EO, primarily eugenol, presents significantly higher EE (78%) compared to cinnamon EO, primarily trans-cinnamaldehyde, (42%), highlighting the influence of chemical profile on complexation behaviour [65]. Eugenol presents a slightly more compact aromatic ring structure, with a hydroxy and methoxy group forming two strong hydrogen bonds with CD [191]. Conversely, trans-cinnamaldehyde has a longer, conjugated aldehyde chain attached to its benzene ring, thus presenting a more elongated structure, which will have more difficulty fitting, as well as only a singular hydrogen bond with the CD complex as opposed to two [192]. Furthermore, in the special case of cinnamon EO, eugenol is also prevalent in relatively large quantities [193], introducing competition for inclusion with trans-cinnamaldehyde in ICs.

### 6.2. Compositional Effects of Essential Oils on Formulation Strategies

**Figure 4:**
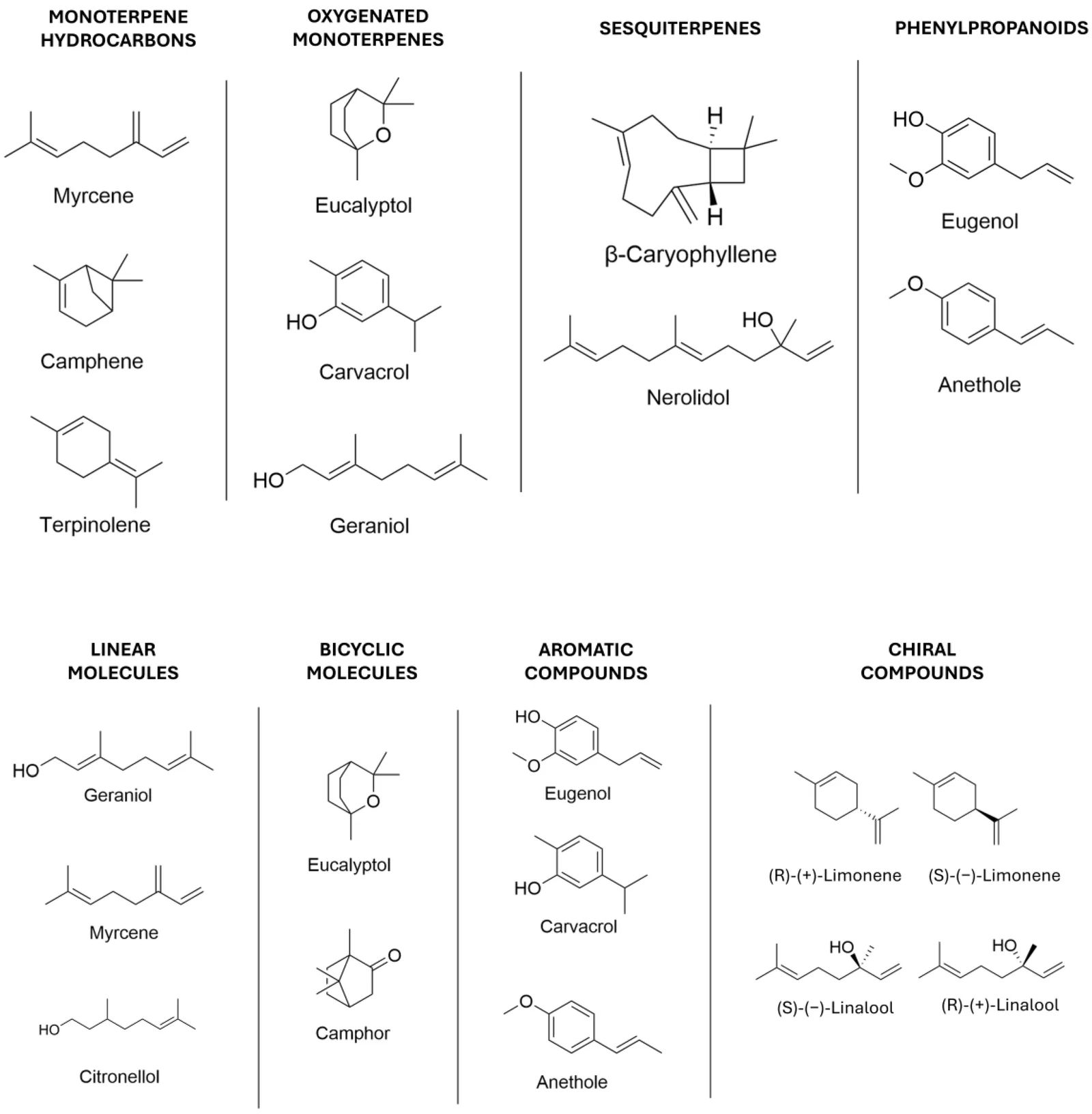
Common chemical classes of EOs and structural considerations of EO molecules. Representative compounds are given.

As touched upon, the behaviour of EOs in formulation systems is governed not only by bulk composition but by the molecular characteristics of individual constituents, including size, polarity, functional groups and three-dimensionality. These factors dictate partitioning, interfacial activity and EE, resulting in selective incorporation as well as heterogenous distribution and release of EO components. The behaviour of EOs in both emulsification and encapsulation systems is fundamentally dictated by the array of chemical classes of their constituent compounds, primarily monoterpene hydrocarbons, oxygenated monoterpenes, sesquiterpenes and phenylpropanoids (Figure 4). Monoterpene hydrocarbons .g., myrcene, camphene, terpinolene), which are small, non-polar and highly volatile, exhibit weak, hydrophobic interactions and tend to remain preferentially in the oil phase of emulsions. Despite their low water solubility, their smaller molecular size relative to sesquiterpenes means they exhibit comparatively greater aqueous phase mobility, which can contribute to Ostwald ripening [37,176]. In contrast, oxygenated monoterpenes (e.g., eucalyptol, geraniol, carvacrol), possess functional groups such as ether, alcohol and phenol, respectively, enabling hydrogen bonding and dipole interactions that promote greater affinity for interfacial regions and carrier matrices, thus enhancing emulsion stability and EE [10,175]. Phenylpropanoids (e.g., eugenol, anethole) exhibit amphiphilic characteristics due to their aromatic ring and polar substituents, allowing them to act as co-surfactants, accumulating at oil–water interfaces and strongly interacting with lipid bilayers or polymer matrices. This can often enhance antimicrobial efficacy but also influences interfacial film properties [76,194]. Similarly, cinnamon EO exhibits relatively hydrophobic interactions due to the high concentration of cinnamaldehyde [115]; however, due to its polar aldehyde moiety, it also facilitates adsorption at oil–water interfaces and interactions with the surfactant system [195,196]. Therefore, cinnamaldehyde can readily partition into lipid membranes, disrupting membrane integrity and contributing substantially to the antimicrobial activity of the EO [197]. Conversely, sesquiterpenes (e.g., β-caryophyllene, α-humulene), which are larger (C_15_) and more hydrophobic than monoterpenes (C_10_), may migrate slowly from the oil phase to the oil –water interface. This will mean that EOs with a higher content of these compounds will contribute little to reducing interfacial tension as opposed to other EOs. Furthermore, many sesquiterpenes display reduced mobility and possess bulky cyclical structures. These rigid molecules pack less efficiently within droplets and lipid assemblies than smaller alicyclic molecules. This can result in larger initial droplet sizes and broader particle distributions. Lastly, their larger molecular volume and higher degree of hydrophobicity increase the likelihood of van der Waals interactions with neighbouring molecules and formulation excipients. This favours retention within the droplet core rather than partitioning towards the interface, decreasing the stabilising contribution of the EO further and increasing the potential for droplet coalescence [76,198]. Collectively, these findings indicate that the relative abundance of each compound class governs not only the physicochemical properties of the EO but also its behaviour during formulation, necessitating composition-specific optimisation strategies.

Beyond broad chemical classification, the molecular structure of individual EO constituents, including size, three-dimensionality, polarity and functional group distribution, plays a critical role in determining their interaction with formulation systems. Molecular size and steric constraints are particularly important in encapsulation processes like CD inclusion, where smaller alicyclic or flexible molecules, e.g., geraniol, myrcene and citronellol, can be readily accommodated within the hydrophobic cavity, whereas bulkier or bicyclic structures, e.g., eucalyptol and camphor, experience steric hindrance that limits complexation efficiency (Figure 4) [66,86]. Similarly, in lipid-based systems such as liposomes and SLNs, molecular geometry influences the extent of insertion into lipid bilayers, with planar or moderately flexible molecules integrating more effectively than rigid bulky compounds, which may disrupt membrane packing and increase vesicle size and PDI [86]. In addition to molecular geometry, the presence of structural features such as aromatic rings further modulates interactions. Aromatic compounds (e.g., eugenol, carvacrol, anethole) exhibit improved intermolecular interactions such as π–π stacking and increased affinity for lipid environments, promoting their localisation at interfaces or within bilayers (Figure 4). The final structural consideration of EO components is chirality. While it has a profound effect on olfactory perception and biological activity (e.g., limonene and linalool), its influence on encapsulation and emulsification behaviour is generally secondary to polarity and functional group chemistry. Only in systems that involve chiral hosts such as CDs does it become important; however, there is little research currently aimed at understanding the interactions of chiral plant metabolites and CDs [199].

**Figure 5:**
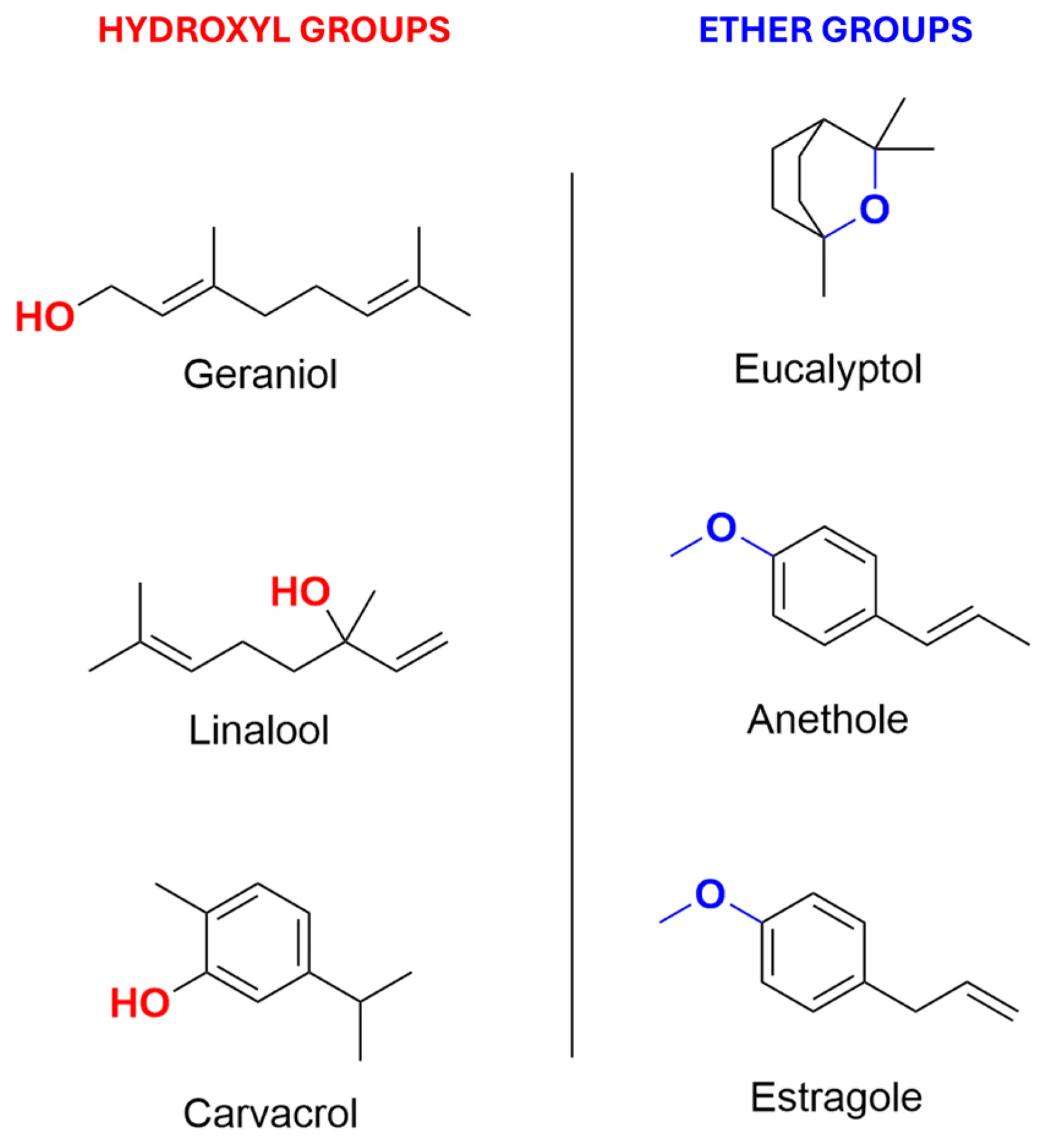
Alcohol and ether-based functional groups of representative EO constituents.

These structural effects act in conjunction with molecular polarity, which ultimately controls partitioning behaviour within formulated systems. Compounds bearing polar functional groups like hydroxyl (e.g., geraniol, linalool, carvacrol) or ether groups (e.g., eucalyptol, anethole, estragole) generally exhibit stronger interactions with carrier matrices through hydrogen bonding and other intermolecular forces, which can influence EE, partitioning behaviour and release kinetics and biological activity [164,200,201] (Figure 5). These structural considerations show that formulation performance cannot be predicted solely from bulk composition but must account for the molecular-level properties of individual constituents, especially those that are more structurally complex.

**Figure 6:**
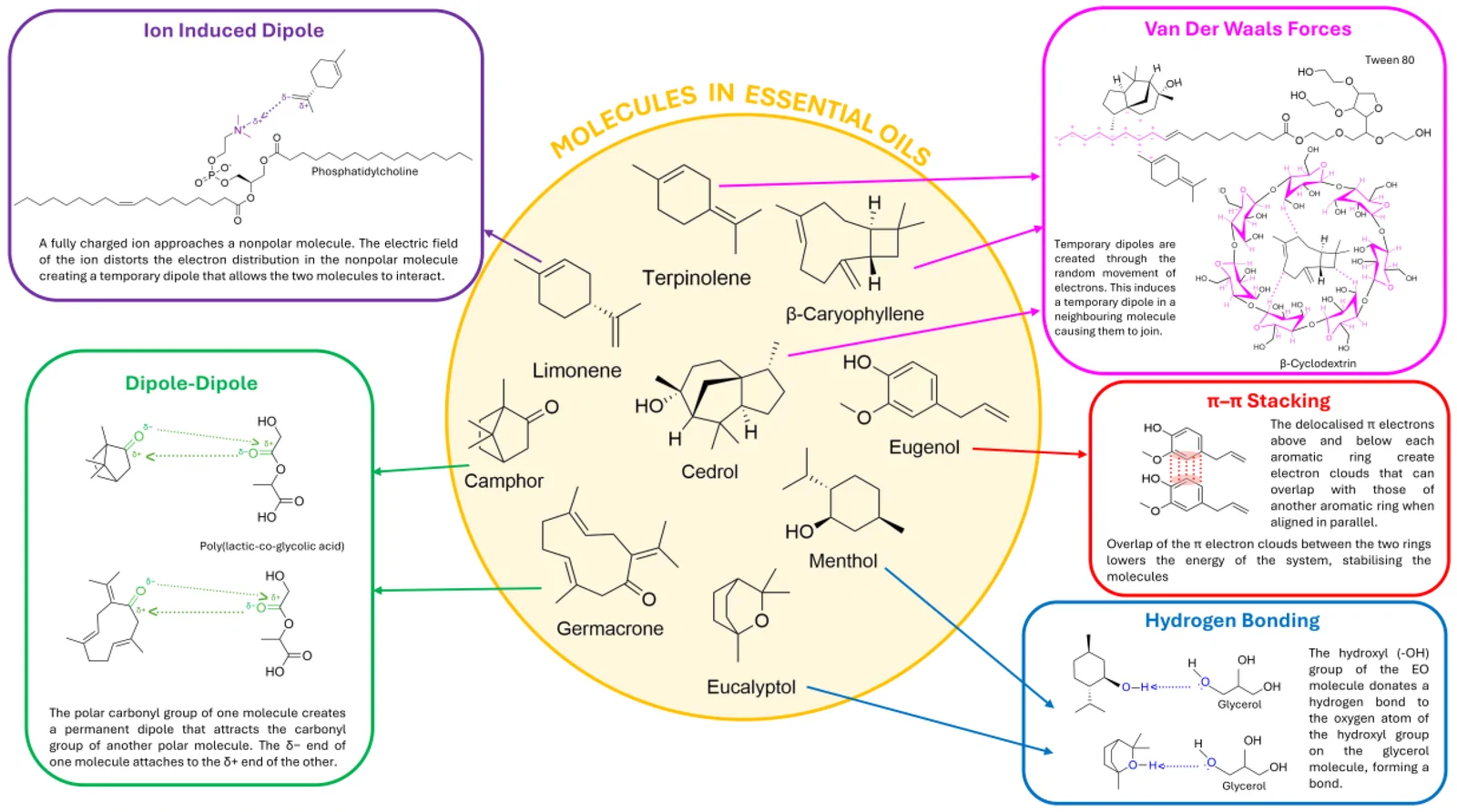
Examples of EO molecules and their types of molecular interactions with formulation excipients.

As molecular complexity increases, so does the nature of intermolecular interactions. At the minimalistic level, weak van der Waals forces are at play with the hydrocarbon based-terpenoid constituents. Inclusion of aromaticity into the EO constituents facilitates a greater degree of π-π interactions, while oxygen functionalisation offers opportunity for hydrogen bonding across both the aliphatic and aromatic-based volatiles (Figure 6). Van der Waals forces represent the baseline interaction that controls the partitioning of non-polar EO compounds, increasing molecular surface and polarisability. This aids in the retention of larger or more hydrophobic structures within lipid or hydrophobic environments [202,203]. Weakly branched alicyclic molecules (e.g., geraniol and linalool) are characterised by high conformational flexibility and accessibly polar functionality, promoting dynamic hydrogen bonding and dipole alignment, which facilitates efficient partitioning into aqueous-interfacial environments and favourable inclusion within CD cavities [87,204]. In contrast, monocyclic and bicyclic systems such as terpinolene and eucalyptol exhibit progressively reduced conformational freedom and increased steric bulk, enhancing hydrophobic dispersion interactions while simultaneously introducing steric penalties that limit formulation approaches like CD complexation and disrupt ordered lipid packing. At higher structural complexity, sesquiterpenes and polycyclic systems further amplify dispersion and entropic contributions due to increased molecular volume and rigidity, often resulting in reduced EE but enhanced retention within hydrophobic lipid domains [66]. Aromatic constituents, like eugenol and anethole, represent a distinct interaction regime in which π-electron density introduces additional π-π stacking and quadrupolar interactions, preferring interfacial localisation and strong affinity for lipid environments (Figure 6) [66,205,206]. Overall, there exists a hierarchy of competing intermolecular forces, whose relative contributions are governed by the traits of EOs and the chosen formulation approach. EOs can rarely be considered as a single functional ingredient but rather are a sum of distinct compounds, which each possess distinctive molecular considerations that must be addressed prior to formulation. During emulsification, EO components partition dynamically according to their physicochemical properties, leading to heterogenous droplet structures in which non-polar hydrocarbons remain in the core, more amphiphilic molecules accumulate at the interface and certain volatile or partially water-soluble compounds may diffuse into the aqueous phase [10,37]. In encapsulated systems like ICs, individual compounds compete for inclusion, favouring those with optimal size and polarity. Whereas in polymeric and lipid-based carriers, differences in affinity for the matrix result in preferential retention of some components and partial loss or surface localisation of others [66,188]. This selective behaviour often leads to a reformulated chemical profile within the delivery system that differs from the original EO composition, as seen with PNs, where more volatile monoterpenes are lost while less volatile components are retained. Nevertheless, synergistic or antagonistic interactions between EO compounds may still occur, especially at interfaces or within lipid environments, influencing both physicochemical stability and biological activity [194,198]. Therefore, EOs in formulation should be regarded as complex dynamic mixtures in which individual molecular behaviour, constituent class and emergent collective effects contribute to overall system performance and selection of the correct formulation excipients.

### 6.3. Safety of Essential Oil Formulations

Although many EOs are recognised as GRAS (Generally Recognised as Safe) for use as flavouring agents, their safety in formulated systems remains less well established. GRAS status applies to specific uses and does not necessarily account for changes in physicochemical properties that occur upon formulation, such as in encapsulated systems. For example, citrus EOs are widely used in emulsified systems to enhance antimicrobial and antioxidant activity; however, incorporation into colloidal structures can alter their bioavailability and biological interactions, meaning that the safety profile of formulated EOs may differ from the free form oil [45]. Likewise, liposomes can interact with biological systems in a size- and composition-dependent manner, influencing cellular uptake, biodistribution and potential toxicity [155]. Their ability to enhance penetration across biological barriers, while advantageous for delivery, necessitates careful evaluation of dose, exposure route and long-term effects. With microemulsions, the high levels of surfactants and co-surfactants required for EO formulations may increase the risk of toxicity, irritation or environmental impact [207]. This was true for a *Forsythia suspensa* (Thunb.) Vahl (golden bell) EO, which proved to require the highest level of surfactant as well as the incorporation of several co-surfactants to generate the most stable formulation [208]. When considering nanoemulsions, on the other hand, their small droplet size can enhance the dispersion and bioavailability of EO components. While this improves functional performance, it may also increase biological exposure and modify absorption pathways [209]. Similarly to surfactants in emulsions, oxidation products of phospholipids, particularly those containing unsaturated fatty acids, and polymers may contribute to cytotoxicity or inflammatory responses [53,155,210]. Additionally, residual solvents from preparation methods or the presence of charged lipids and stabilising agents may influence biocompatibility. While liposomes, SLNs and NLCs are generally less reliant on high surfactant concentrations compared with emulsions, the inclusion of certain additives (e.g., ethanol) may introduce additional safety concerns [50]. With ICs, β-CDs and HP-β-CDs are widely accepted for food and pharmaceutical use, due to their carbohydrate-based structure. Nevertheless, maximum permitted uptake levels, degree of substitution (for modified CDs), and potential gastrointestinal effects at high concentrations must be considered. Native β-CDs have limited systemic absorption and may accumulate in the gastrointestinal tract at high concentrations, potentially leading to osmotic effects such as diarrhoea [211]. So, where increased concentration of CDs leads to better EE for EOs, this could have some safety implications.

These considerations are particularly relevant given the increasing regulatory attention surrounding nanoscale delivery systems. Due to their small size, high surface area and increased solubility, such systems may exhibit distinct biological interactions, bioavailability and toxicity profiles compared to bulk materials (Table 1) [99,135,212,213,214].

**Table 1:** Summary of the advantages and limitations presented by each formulation approach.

Formulation Approach	Structure	Advantages	Problems
Microemulsions	Thermodynamically stable mixtures of oil, water and surfactant. Spontaneously formed.	- Spontaneously solubilise hydrophobic EO constituents in water without high-energy input [39]. - Thermodynamic stability minimises phase separation during storage, maintaining EO dispersion. - High interfacial area enhances activity. - Suitable for incorporating amphiphilic terpenoids (e.g., linalool) that can partition at the oil–water interface [40].	- High surfactant concentrations may be required, limiting food and pharmaceutical applications, due to toxicity concerns [207]. - Surfactants may alter the sensory characteristics (taste, odour) of EO-containing formulations [41]. - Limited protection against post-application volatilisation compared with encapsulated systems.
Nanoemulsions	Kinetically stable dispersions of nanoscale oil droplets in water. Formed using external energy.	- Improved dispersion of poorly water-soluble EOs in aqueous formulations [39]. - Small droplet size increases surface area, enhancing bioactivity and interaction with biological membranes. - Require lower surfactant levels than microemulsions [39]. - Well established for food flavourings, edible coatings and pest and crop management [9,42,43,44,45,46].	- Susceptible to coalescence and Ostwald ripening over time [41]. - Volatile terpenes may gradually diffuse from droplets during storage. - High-energy preparation methods can cause loss of highly volatile constituents [39]. - Provide less protection against oxidation and volatilisation than matrix-based encapsulation systems.
Vesicular Systems	Vesicles composed of one or more phospholipid bilayers surrounding an aqueous core.	- Phospholipid bilayers protect against EO oxidation and volatilisation. - Hydrophobic EO constituents can partition into the lipid membrane, improving retention [48,50]. - Attractive for topical, mucosal and pharmaceutical EO delivery. - Reduction in irritation associated with direct contact of EOs. - Dermal penetration improved.	- Some EO components disrupt membrane integrity, causing leakage [79,81,82]. - Unsaturated phospholipids are prone to oxidation [157,158]. - EE is limited for highly volatile EO constituents. - Costly materials hinder large-scale production.
Lipid Carriers	Solid or semi-solid lipid core stabilised by surfactants. EOs and their compounds are trapped within the lipid matrix.	- Particularly suitable for preserving terpenes. - Provide sustained release, prolonging bioactivity. - NLCs offer higher EO loading and retention due to their imperfect crystal structure [54,55]. - Improve bioavailability and residence time in topical and food-packaging applications [60].	- Preparation requires elevated temperatures, so EOs may degrade [56,57]. - Expulsion during storage [47,53,89]. - Specialised lipids and processing equipment may increase costs.
PNs	Nanoparticles formed from biodegradable polymers such as chitosan, PLGA and PVA, with EO entrapped.	- Protection against oxidation, photodegradation and evaporation. - Sustained and targeted release. - Surface modification enables targeting of biological tissues, biofilms and insect pests [61].	- EE and release may vary dependent on EO complexity. - Residual solvents or processing conditions can affect EO constituents [135]. - Polymer interaction with EOs components reduces release rates [164,165]. - Regulatory acceptance is challenging [131]. - Residual oil can aggregate on PN surfaces, causing instability [185].
ICs	Molecular encapsulation where EO components are hosted within the hydrophobic cavity of cyclodextrins via non-covalent interactions.	- Effective at stabilising volatile monoterpenes and phenolic constituents [49,99]. - Mask strong EO odours and flavours [65,66]. - Increase water solubility of some EO components. - Applications in food and biopesticides in agricultural sectors [13,65,206]. - Prepared under mild conditions [49,100].	- Cavities only accommodate molecules within a specific size range [66]. - EOs cannot be wholly complexed [65]. - EE is higher for individual compounds rather than whole EOs. - Competitive binding between EO constituents may reduce complexation of target compounds [65]. - Lower loading capacity.

## 7. Conclusions and Future Directions

Emulsification and encapsulation technologies constitute the primary formulation approaches for enhancing the physicochemical stability, bioavailability and functional performance of EOs. Emulsion-based systems offer advantages in terms of simplicity, scalability and high loading capacity, enabling improved aqueous dispersibility and rapid bioavailability. However, these systems often require high surfactant concentrations, offer limited control over sustained release and give reduced long-term protection against volatilisation and oxidative degradation. Encapsulation-based systems provide more structurally defined routes to EO delivery, with high biocompatibility, controlled release and improved structural integrity. However, selective incorporation in the presence of complex EOs, solvent-related toxicity and reduced loading capacity are important considerations when using encapsulation as the selected approach. Overall, no single primary delivery system can be deemed universally optimal. Instead, the selection of an appropriate formulation strategy must be guided by the characteristics of the EO itself, the intended application and the desired release profile. In food applications, the chosen formulation must improve the aqueous dispersibility and stability of an EO while maintaining sensory acceptability and complying with food safety requirements. In this area, nanoemulsions, microemulsions and CDs have received considerable attention due to their ability to enhance dispersion and protect volatile constituents. In pharmaceutical and biomedical applications, greater emphasis is placed on biocompatibility, controlled release, tissue penetration and reproducibility, driving interest in vesicular systems, lipid carriers and PNs. Conversely, agricultural applications require cost-effective systems capable of protecting EO actives from environmental degradation and extending field persistence, leading to increased exploration of polymer-based carriers, ICs and alternative encapsulation technologies suitable for large-scale deployment. Therefore, formulation selection cannot be considered independently of EO composition and intended use, as the optimal delivery strategy requires balancing the physicochemical properties of the oil with application-specific performance requirements.

**Figure 7:**
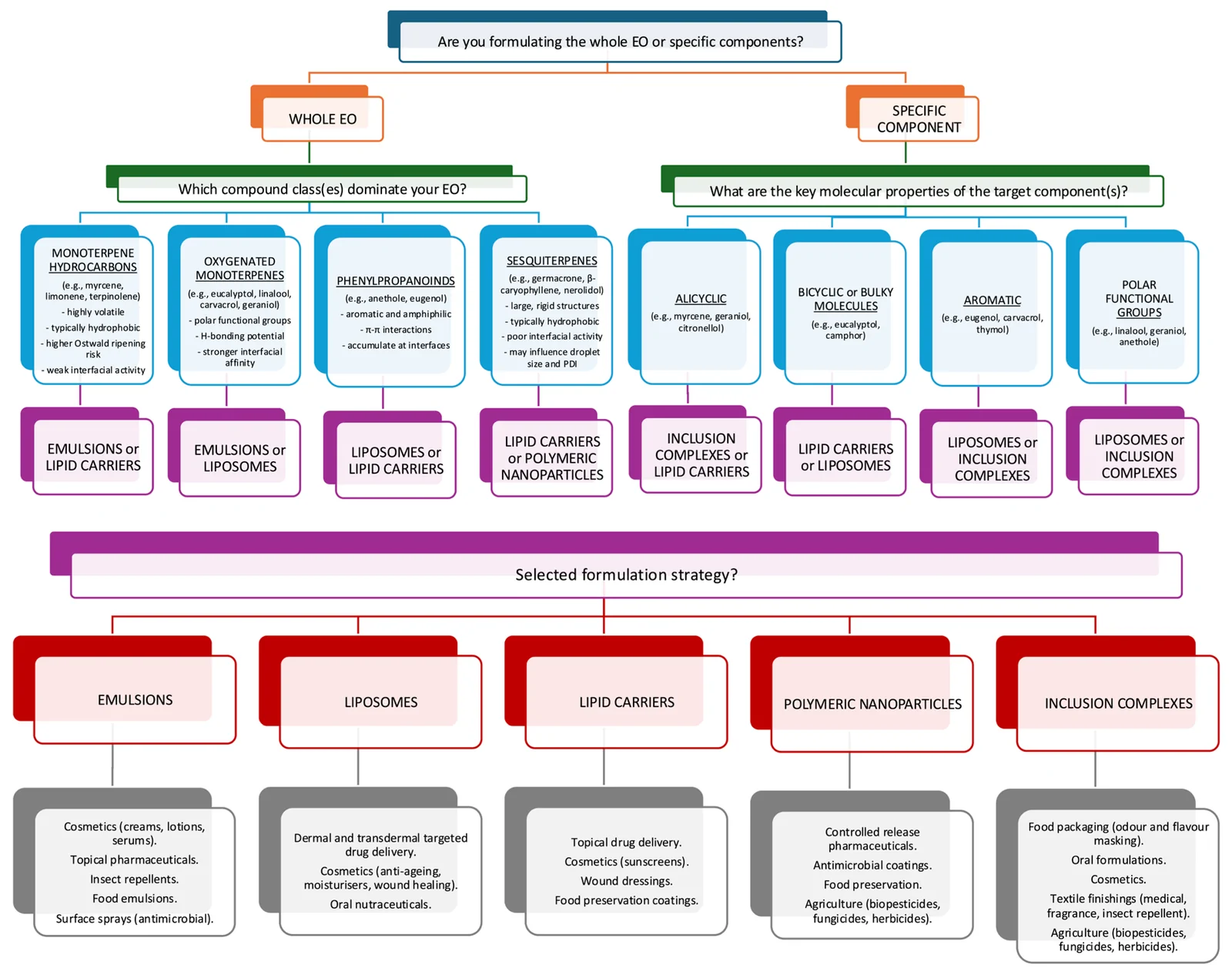
Decision framework for selecting formulation strategies for essential oils based on their chemical composition and targeted application.

Looking forward, progress will depend not only on the discovery of new bioactive EOs and development of more rational, application-specific strategies but also on a more holistic understanding of EOs as multicomponent mixtures rather than single active ingredients. Formulation strategies frequently centre the major constituents of an EO, with reports measuring the EE and controlled release on the basis of these specific components. Not only are the interactions between formulation excipients and minor constituents often overlooked, but the consequences of these interactions for EE, release behaviour and, ultimately, biological activity are also rarely considered. Differential encapsulation or release of individual constituents may alter the chemical composition of the formulation, potentially disrupting the synergistic interactions responsible for the biological activity of the original EO. Furthermore, the chemical composition of EOs is inherently variable, being influenced by factors such as plant genotype, geographical origin, environmental and seasonal conditions, cultivation practices and extraction methodology. This compositional variability can significantly alter physicochemical properties, biological efficacy and formulation behaviour, yet it is often underappreciated during formulation development. Future research should therefore place greater emphasis on comprehensive chemical characterisation and batch-to-batch standardisation, enabling formulation design to be tailored to the complete chemical profile of an EO (Figure 7). Coupling this with advances in analytical characterisation, predictive formulation design and scalable manufacturing would enable more precise matching of delivery systems to the physicochemical properties of individual oils and their intended applications. Greater emphasis on sustainable, food-grade and biodegradable carrier materials, together with harmonised regulatory frameworks and standardised methods for evaluating formulation performance, will be critical in accelerating the commercial viability of EOs in formulation. Ultimately, the future of EO formulation lies not in identifying a universally superior delivery platform but in developing chemically informed, application-driven formulation strategies. This will, in turn, maximise stability, bioavailability and functional performance while meeting the practical demands of industrial scalability and regulatory approval.

## Data Availability

No data were used for the research described in this article.

## Abbreviations

CD	cyclodextrin
DOPC	dioleoylphosphatidylcholine
DOPE	dioleoylphosphatidylethanolamine
EE	encapsulation efficiency
EIC	emulsion inversion point
EO	essential oil
FDA	Food and Drug Administration
GRAS	generally recognised as safe
HEM	high energy method
HLB	hydrophilic–lipophilic balance
HPH	high-pressure homogenisation
HP-β-CD	hydroxypropyl-β-cyclodextrin
HSH	high-shear homogenisation
IC	inclusion complex
LEM	low energy method
LUV	large unilamellar vesicle
MLV	multilamellar vesicle
MOF	metal organic framework
NLC	nanostructured lipid carrier
O/W	oil-in-water
PC	phosphatidylcholine
PDI	polydispersity index
PE	phosphatidylethanolamine
PEG	polyethylene glycol
PIC	phase inversion composition
PIT	phase inversion temperature
PLGA	poly(lactic-co-glycolic) acid
PN	polymeric nanoparticle
PVA	polyvinyl alcohol
SLN	solid lipid nanoparticle
SUV	small unilamellar vesicle
T_g_	glass transition temperature
W/O	water-in-oil

## References

[1] Baptista-Silva S., Borges S., Ramos O.L., Pintado M., Sarmento B. (2020). The progress of essential oils as potential therapeutic agents: A review. J. Essent. Oil Res..

[2] Javed S., Mangla B., Salawi A., Sultan M.H., Almoshari Y., Ahsan W. (2024). Essential Oils as Dermocosmetic Agents, Their Mechanism of Action and Nanolipidic Formulations for Maximized Skincare. Cosmetics.

[3] Sarmah K., Anbalagan T., Marimuthu M., Mariappan P., Angappan S., Vaithiyanathan S. (2025). Innovative formulation strategies for botanical-and essential oil-based insecticides. J. Pest Sci..

[4] Pavoni L., Perinelli D.R., Bonacucina G., Cespi M., Palmieri G.F. (2020). An Overview of Micro- and Nanoemulsions as Vehicles for Essential Oils: Formulation, Preparation and Stability. Nanomaterials.

[5] Manconi M., Petretto G., D’hallewin G., Escribano E., Milia E., Pinna R., Palmieri A., Firoznezhad M., Peris J.E., Usach I. (2018). *Thymus* essential oil extraction, characterization and incorporation in phospholipid vesicles for the antioxidant/antibacterial treatment of oral cavity diseases. Colloids Surf. B Biointerfaces.

[6] Sinico C., De Logu A., Lai F., Valenti D., Manconi M., Loy G., Bonsignore L., Fadda A.M. (2005). Liposomal incorporation of *Artemisia arborescens* L. essential oil and in vitro antiviral activity. Eur. J. Pharm. Biopharm..

[7] Adeyemi S.B., Akere A.M., Orege J.I., Ejeromeghene O., Orege O.B., Akolade J.O. (2023). Polymeric nanoparticles for enhanced delivery and improved bioactivity of essential oils. Heliyon.

[8] Wadhwa G., Kumar S., Chhabra L., Mahant S., Rao R. (2017). Essential oil–cyclodextrin complexes: An updated review. J. Incl. Phenom. Macrocycl. Chem..

[9] Bhargava K., Conti D.S., da Rocha S.R.P., Zhang Y. (2015). Application of an oregano oil nanoemulsion to the control of foodborne bacteria on fresh lettuce. Food Microbiol..

[10] Donsì F., Annunziata M., Sessa M., Ferrari G. (2011). Nanoencapsulation of essential oils to enhance their antimicrobial activity in foods. LWT-Food Sci. Technol..

[11] Dupuis V., Cerbu C., Witkowski L., Potarniche A.-V., Timar M.C., Żychska M., Sabliov C.M. (2022). Nanodelivery of essential oils as efficient tools against antimicrobial resistance: A review of the type and physical-chemical properties of the delivery systems and applications. Drug Deliv..

[12] Rejinold N.S., Muthunarayanan M., Muthuchelian K., Chennazhi K.P., Nair S.V., Jayakumar R. (2011). Saponin-loaded chitosan nanoparticles and their cytotoxicity to cancer cell lines in vitro. Carbohydr. Polym..

[13] Maes C., Bouquillon S., Fauconnier M.-L. (2019). Encapsulation of Essential Oils for the Development of Biosourced Pesticides with Controlled Release: A Review. Molecules.

[14] Niza G.E., Rubio M.A., Ahrazem el Kadiri O., Gomez Gomez L., Martinez L.A., Gomez L.P. (2022). Chitosan Nanoparticles Containing Encapsulated Garlic Essential Oil (*Allium sativum* L.) and Use Thereof. European Patent.

[15] Russo R., Mizoguchi H., Watanabe C., Hamamura K., Katsuyama S., Komatsu T., Morrone L.A., Adornetto A., Lagana A.S., Scuteri D. (2022). Nanotechnology-Based Delivery System of Bergamot Essential Oil, Method of Preparation of the System and Uses Thereof. U.S. Patent.

[16] Shen J., Su Y., Liu Y. (2022). Application of *Litsea cubeba* Essential Oil Liposome in Anti-Inflammation and Acne Removal. Chinese Patent.

[17] Shi C., Li W., Huang C., Fang D., Zhou A., Lu T., Wang J. (2022). Nanometer Film Added with Oregano Essential Oil Cyclodextrin Inclusion Compound and Preparation Method and Application Thereif. Chinese Patent.

[18] Sun Y., Xie L., Yang X., Gan H., Chen L. (2022). A Kind of Mustard Essential Oil Liposome and Preparation Method and Application Thereof. Chinese Patent.

[19] Tosun F., Demirci B., Demirci F., Karadag A.E., Üstundag O.N. (2021). Development of Microemulsion Formulation from *Rosmarinus officinalis* Essential Oil and the Antifungal Effect of These Formulations. European Patent.

[20] Wang Y., Fu L., Chen J., Wang F. (2020). Preparation Method of *Radix chinensis* Essential Oil Nanoemulsion and Its Application in Salmon Fresh-Keeping. Chinese Patent.

[21] Wang Y., Zhang S., Wang X., Fan Y., Gai C., Li L., Wang S.C., Ye H. (2024). Garlic Essential Oil Antibacterial Microemulsion for Shrimp Farming, Preparation Method and Application. Chinese Patent.

[22] Zhu X., Wang Y., Shan Y., Li G., Fu F., Su D., Tao N., Gong L., Ding S. (2021). *Litsea cubeba* Essential Oil-Hydroxypropyl-Beta-Cyclodextrin Inclusion Compound and Preparation Method and Application Thereof. Chinese Patent.

[23] Research E. Mevalone. https://www.edenresearch.com/products/mevalone.aspx.

[24] Eastman (2021). Technical Data Sheet: Cedroz.

[25] Tayeb H.H., Felimban R., Hasaballah N., Bin Mahfouz J., Chaudhary A., Felemban M., Alnadwi F., Rizg W. (2022). Essential Oil Nanoemulsion and Methods of Use Thereof. U.S. Patent.

[26] Kumosani T., Barbour E., Werner K., Yaghmoor S. (2021). Method for Preparing Liposomes Containing an Essential Oil in an Oil-In-Water Emulsion. U.S. Patent.

[27] Tagra CelluOil. https://tagra.com/celluoil/#product-detail.

[28] Biochemistry M. RootBioTec™ HW. https://www.mibellebiochemistry.com/rootbiotectm-hw.

[29] Ez-zoubi A., Annemer S., El Amrani S., Ez zoubi Y., Farah A. (2023). Encapsulation of *Origanum compactum* essential oil in beta-cyclodextrin metal organic frameworks: Characterization, optimization, and antioxidant activity. J. Food Process. Preserv..

[30] Motelica L., Ficai D., Petrisor G., Oprea O.-C., Trușcǎ R.-D., Ficai A., Andronescu E., Hudita A., Holban A.M. (2024). Antimicrobial hydroxyethyl-cellulose-based composite films with zinc oxide and mesoporous silica loaded with cinnamon essential oil. Pharmaceutics.

[31] Ebadollahi A., Sendi J.J., Aliakbar A. (2017). Efficacy of nanoencapsulated *Thymus eriocalyx* and *Thymus kotschyanus* essential oils by a mesoporous material MCM-41 against *Tetranychus urticae* (Acari: Tetranychidae). J. Econ. Entomol..

[32] Kiliç D., Kutlu E., Muhammad Asım A., Emen F.M., Demirdöğrn R.E. (2022). Preparation of MCM-48 loaded with lavender oil and vitamin E under supercritical carbondioxide conditions and its usein peel formulation. J. Nat. Sci. Technol..

[33] Weisany W., Samadi S., Tahir N.A., Amini J., Hossaini S. (2022). Nano-encapsulated with mesoporous silica enhanced the antifungal activity of essential oil against *Botrytis cinerea* (Helotiales; Sclerotiniaceae) and *Colletotrichum nymphaeae* (Glomerellales; Glomerellaceae). Physiol. Mol. Plant Pathol..

[34] Angeli G.K., Kotzabasaki M.I., Maraveas C. (2025). Metal organic frameworks for smart storage and delivery of aromatic volatiles and essential oils in agrifood. Appl. Sci..

[35] Li X., Huang L., Wang Y., Zhang M., Han B., Huang X., Ge Y. (2026). The volatilization stability, mechanical property and sustained-antibacterial effect of solidified *Artemisia argyi* leaf essential oil by porous carriers. Ind. Crops Prod..

[36] Wang Y., Du Y.-T., Xue W.-Y., Wang L., Li R., Jiang Z.-T., Tang S.-H., Tan J. (2023). Enhanced preservation effects of clove (*Syzygium aromaticum*) essential oil on the processing of Chinese bacon (preserved meat products) by beta cyclodextrin metal organic frameworks (β-CD-MOFs). Meat Sci..

[37] McClements D.J. (2004). Food Emulsions: Principles, Practices, and Techniques.

[38] Anton N., Vandamme T.F. (2011). Nano-emulsions and micro-emulsions: Clarifications of the critical differences. Pharm. Res..

[39] McClements D.J. (2012). Nanoemulsions versus microemulsions: Terminology, differences, and similarities. Soft Matter.

[40] Langevin D. (1988). Microemulsions. Acc. Chem. Res..

[41] Trujillo-Cayado L.A., Santos J., Calero N., Alfaro-Rodríguez M.C., Muñoz J. (2020). Strategies for reducing Ostwald ripening phenomenon in nanoemulsions based on thyme essential oil. J. Sci. Food Agric..

[42] Campana R., Tiboni M., Maggi F., Cappellacci L., Cianfaglione K., Morshedloo M.R., Frangipani E., Casettari L. (2022). Comparative Analysis of the Antimicrobial Activity of Essential Oils and Their Formulated Microemulsions against Foodborne Pathogens and Spoilage Bacteria. Antibiotics.

[43] Gharenaghadeh S., Karimi N., Forghani S., Nourazarian M., Gharehnaghadeh S., Kafil H.S. (2017). Application of *Salvia multicaulis* essential oil-containing nanoemulsion against food-borne pathogens. Food Biosci..

[44] Grifoni L., Sacco C., Donato R., Tziakas S., Tomou E.-M., Skaltsa H., Vanti G., Bergonzi M.C., Bilia A.R. (2024). Environmentally Friendly Microemulsions of Essential Oils of *Artemisia annua* and *Salvia fruticosa* to Protect Crops against *Fusarium verticillioides*. Nanomaterials.

[45] Medeleanu M.L., Fărcaș A.C., Coman C., Leopold L., Diaconeasa Z., Socaci S.A. (2023). Citrus essential oils—Based nano-emulsions: Functional properties and potential applications. Food Chem. X.

[46] Mendes J.F., Norcino L.B., Martins H.H.A., Manrich A., Otoni C.G., Carvalho E.E.N., Piccoli R.H., Oliveira J.E., Pinheiro A.C.M., Mattoso L.H.C. (2020). Correlating emulsion characteristics with the properties of active starch films loaded with lemongrass essential oil. Food Hydrocoll..

[47] Yammine J., Chihib N.-E., Gharsallaoui A., Ismail A., Karam L. (2024). Advances in essential oils encapsulation: Development, characterization and release mechanisms. Polym. Bull..

[48] Sherry M., Charcosset C., Fessi H., Greige-Gerges H. (2013). Essential oils encapsulated in liposomes: A review. J. Liposome Res..

[49] Marques H.M.C. (2010). A review on cyclodextrin encapsulation of essential oils and volatiles. Flavour Fragr. J..

[50] Ricci A., Stefanuto L., Gasperi T., Bruni F., Tofani D. (2024). Lipid Nanovesicles for Antioxidant Delivery in Skin: Liposomes, Ufasomes, Ethosomes, and Niosomes. Antioxidants.

[51] Voinea M., Simionescu M. (2002). Designing of ‘intelligent’ liposomes for efficient delivery of drugs. J. Cell. Mol. Med..

[52] Baldim I., Oliveira A.M., Souto E.B., Oliveira W.P. (2022). Cyclodextrins-in-Liposomes: A Promising Delivery System for *Lippia sidoides* and *Syzygium aromaticum* Essential Oils. Life.

[53] Müller R.H., Mäder K., Gohla S. (2000). Solid lipid nanoparticles (SLN) for controlled drug delivery—A review of the state of the art. Eur. J. Pharm. Biopharm..

[54] Müller R.H., Radtke M., Wissing S.A. (2002). Nanostructured lipid matrices for improved microencapsulation of drugs. Int. J. Pharm..

[55] Pardeike J., Hommoss A., Müller R.H. (2009). Lipid nanoparticles (SLN, NLC) in cosmetic and pharmaceutical dermal products. Int. J. Pharm..

[56] Fathi M., Vinceković M., Jurić S., Viskić M., Režek Jambrak A., Donsì F. (2021). Food-grade colloidal systems for the delivery of essential oils. Food Rev. Int..

[57] Nair A., Mallya R., Suvarna V., Khan T.A., Momin M., Omri A. (2022). Nanoparticles-Attractive Carriers of Antimicrobial Essential Oils. Antibiotics.

[58] Carbone C., Teixeira M.d.C., Sousa M.d.C., Martins-Gomes C., Silva A.M., Souto E.M.B., Musumeci T. (2019). Clotrimazole-loaded mediterranean essential oils NLC: A synergic treatment of Candida skin infections. Pharmaceutics.

[59] Nasseri M., Golmohammadzadeh S., Arouiee H., Jaafari M.R., Neamati H. (2016). Antifungal activity of *Zataria multiflora* essential oil-loaded solid lipid nanoparticles in-vitro condition. Iran. J. Basic Med. Sci..

[60] Mehran M., Masoum S., Memarzadeh M. (2020). Microencapsulation of *Mentha spicata* essential oil by spray drying: Optimization, characterization, release kinetics of essential oil from microcapsules in food models. Ind. Crops Prod..

[61] Shetta A., Ali I.H., Sharaf N.S., Mamdouh W. (2024). Review of strategic methods for encapsulating essential oils into chitosan nanosystems and their applications. Int. J. Biol. Macromol..

[62] Almeida K.B., Ramos A.S., Nunes J.B.B., Silva B.O., Ferraz E.R.A., Fernandes A.S., Felzenszwalb I., Amaral A.C.F., Roullin V.G., Falcão D.Q. (2019). PLGA nanoparticles optimized by Box-Behnken for efficient encapsulation of therapeutic *Cymbopogon citratus* essential oil. Colloids Surf. B Biointerfaces.

[63] Rajkumar V., Gunasekaran C., Paul C.A., Dharmaraj J. (2020). Development of encapsulated peppermint essential oil in chitosan nanoparticles: Characterization and biological efficacy against stored-grain pest control. Pestic. Biochem. Physiol..

[64] Procopio F.R., Brexó R.P., Vitolano L.E.S., Martins M.E.d.M., Astolfo M.E.d.A., Bogusz Junior S., Ferreira M.D. (2024). Development of essential oils inclusion complexes: A nanotechnology approach with enhanced thermal and light stability. Discov. Nano.

[65] Hill L.E., Gomes C., Taylor T.M. (2013). Characterization of beta-cyclodextrin inclusion complexes containing essential oils (trans-cinnamaldehyde, eugenol, cinnamon bark, and clove bud extracts) for antimicrobial delivery applications. Food Sci. Technol..

[66] Kfoury M., Auezova L., Greige-Gerges H., Fourmentin S. (2015). Promising applications of cyclodextrins in food: Improvement of essential oils retention, controlled release and antiradical activity. Carbohydr. Polym..

[67] Yamashita Y., Miyahara R., Sakamoto K. (2017). Emulsion and emulsification technology. Cosmet. Sci. Technol..

[68] Kralova I., Sjöblom J. (2009). Surfactants used in food industry: A review. J. Dispers. Sci. Technol..

[69] Chang Y., McClements D.J. (2014). Optimization of orange oil nanoemulsion formation by isothermal low-energy methods: Influence of the oil phase, surfactant, and temperature. J. Agric. Food Chem..

[70] Nirmal N.P., Mereddy R., Li L., Sultanbawa Y. (2018). Formulation, characterisation and antibacterial activity of lemon myrtle and anise myrtle essential oil in water nanoemulsion. Food Chem..

[71] Balasubramani S., Rajendhiran T., Moola A.K., Diana R.K.B. (2017). Development of nanoemulsion from *Vitex negundo* L. essential oil and their efficacy of antioxidant, antimicrobial and larvicidal activities (*Aedes aegypti* L.). Environ. Sci. Pollut. Res..

[72] Sugumar S., Clarke S.K., Nirmala M.J., Tyagi B.K., Mukherjee A., Chandrasekaran N. (2014). Nanoemulsion of eucalyptus oil and its larvicidal activity against *Culex quinquefasciatus*. Bull. Entomol. Res..

[73] Alonso L., Cardoso É.J.S., Gomes R.S., Mendanha S.A., Dorta M.L., Alonso A. (2019). Antileishmanial and cytotoxic activities of ionic surfactants compared to those of miltefosine. Colloids Surf. B Biointerfaces.

[74] Syed H.K., Peh K.K. (2014). Identification of phases of various oil, surfactant/co-surfactants and water system by ternary phase diagram. Acta Pol. Pharm..

[75] Wang Y., Zhou K., Ye C., Shang Y., Wang F. (2025). Preparation and characterization of microemulsions formulated with PEG fatty acid glycerides. Colloids Surf. B Biointerfaces.

[76] Campolo O., Giunti G., Laigle M., Michel T., Palmeri V. (2020). Essential oil-based nano-emulsions: Effect of different surfactants, sonication and plant species on physicochemical characteristics. Ind. Crops Prod..

[77] Golwala P., Rathod S., Patil R., Joshi A., Ray D., Aswal V.K., Bahadur P., Tiwari S. (2020). Effect of cosurfactant addition on phase behavior and microstructure of a water dilutable microemulsion. Colloids Surf. B Biointerfaces.

[78] Bouton F. (2010). Influence of Terpenes and Terpenoids on the Phase Behavior of Micro-and Macro-Emulsions. Ph.D. Thesis.

[79] Torchilin V.P. (2005). Recent advances with liposomes as pharmaceutical carriers. Nat. Rev. Drug Discov..

[80] McClements D.J. (2018). Encapsulation, protection, and delivery of bioactive proteins and peptides using nanoparticle and microparticle systems: A review. Adv. Colloid Interface Sci..

[81] Lasic D.D. (1993). Liposomes: From Physics to Applications.

[82] Sheikholeslami B., Lam N.W., Dua K., Haghi M. (2022). Exploring the impact of physicochemical properties of liposomal formulations on their in vivo fate. Life Sci..

[83] FDA (2018). Liposome Drug Products: Chemistry, Manufacturing, and Controls; Human Pharmacokinetics and Bioavailability; and Labeling Documentation.

[84] Zhu R., Morkos B., Liu L. (2026). Therapeutic Potentials and Encapsulation Strategies of Essential Oils. Processes.

[85] Wang J.-P., Huang Z.-R., Zhang C., Ni Y.-R., Li B.-T., Wang Y., Wu J.-F. (2025). Methodological advances in liposomal encapsulation efficiency determination: Systematic review and analysis. J. Drug Target..

[86] Sebaaly C., Jraij A., Fessi H., Charcosset C., Greige-Gerges H. (2015). Preparation and characterization of clove essential oil-loaded liposomes. Food Chem..

[87] Turina A.d.V., Nolan M.V., Zygadlo J.A., Perillo M.A. (2006). Natural terpenes: Self-assembly and membrane partitioning. Biophys. Chem..

[88] Üner M. (2006). Preparation, characterization and physico-chemical properties of solid lipid nanoparticles (SLN) and nanostructured lipid carriers (NLC): Their benefits as colloidal drug carrier systems. Die Pharm.-Int. J. Pharm. Sci..

[89] Mehnert W., Mäder K. (2012). Solid lipid nanoparticles: Production, characterization and applications. Adv. Drug Deliv. Rev..

[90] Siepmann J., Peppas N.A. (2012). Modeling of drug release from delivery systems based on hydroxypropyl methylcellulose (HPMC). Adv. Drug Deliv. Rev..

[91] Zielińska A., Ferreira N.R., Feliczak-Guzik A., Nowak I., Souto E.B. (2020). Loading, release profile and accelerated stability assessment of monoterpenes-loaded solid lipid nanoparticles (SLN). Pharm. Dev. Technol..

[92] De R., Mahata M.K., Kim K.-T. (2022). Structure-Based Varieties of Polymeric Nanocarriers and Influences of Their Physicochemical Properties on Drug Delivery Profiles. Adv. Sci..

[93] Yuan M., Siqi C., Ping L., Yezheng H., Fang C., Yifan C., Xianqin Y. (2023). Gelatin Improves the Performance of Oregano Essential Oil Nanoparticle Composite Films—Application to the Preservation of Mullet. Foods.

[94] Dima C., Dima S. (2015). Essential oils in foods: Extraction, stabilization, and toxicity. Curr. Opin. Food Sci..

[95] Gasmi H., Danede F., Siepmann J., Siepmann F. (2015). Does PLGA microparticle swelling control drug release? New insight based on single particle swelling studies. J. Control. Release.

[96] Lappe S., Mulac D., Langer K. (2017). Polymeric nanoparticles—Influence of the glass transition temperature on drug release. Int. J. Pharm..

[97] Stiepel R.T., Pena E.S., Ehrenzeller S.A., Gallovic M.D., Lifshits L.M., Genito C.J., Bachelder E.M., Ainslie K.M. (2022). A predictive mechanistic model of drug release from surface eroding polymeric nanoparticles. J. Control. Release.

[98] Imel A.E., Rostom S., Holley W., Baskaran D., Mays J.W., Dadmun M.D. (2017). The tracer diffusion coefficient of soft nanoparticles in a linear polymer matrix. R. Soc. Chem. Adv..

[99] Del Valle E.M.M. (2004). Cyclodextrins and their uses: A review. Process Biochem..

[100] Loftsson T., Brewster M.E. (2010). Pharmaceutical applications of cyclodextrins: Basic science and product development. J. Pharm. Pharmacol..

[101] Poulson B.G., Alsulami Q.A., Sharfalddin A., El Agammy E.F., Mouffouk F., Emwas A.-H., Jaremko L., Jaremko M. (2022). Cyclodextrins: Structural, Chemical, and Physical Properties, and Applications. Polysaccharides.

[102] Jambhekar S.S., Breen P. (2016). Cyclodextrins in pharmaceutical formulations I: Structure and physicochemical properties, formation of complexes, and types of complex. Drug Discov. Today.

[103] Sakulku U., Nuchuchua O., Uawongyart N., Puttipipatkhachorn S., Soottitantawat A., Ruktanonchai U. (2009). Characterization and mosquito repellent activity of citronella oil nanoemulsion. Int. J. Pharm..

[104] Xing Z., Xu Y., Feng X., Gao C., Wu D., Cheng W., Meng L., Wang Z., Xu T., Tang X. (2024). Fabrication of cinnamon essential oil nanoemulsions with high antibacterial activities via microfluidization. Food Chem..

[105] Hashtjin A.M., Abbasi S. (2015). Nano-emulsification of orange peel essential oil using sonication and native gums. Food Hydrocoll..

[106] Borrin T.R., Georges E.L., Moraes I.C.F., Pinho S.C. (2016). Curcumin-loaded nanoemulsions produced by the emulsion inversion point (EIP) method: An evaluation of process parameters and physico-chemical stability. J. Food Eng..

[107] Feng J., Rodríguez-Abreu C., Esquena J., Solans C. (2020). A concise review on nano-emulsion formation by the phase inversion composition (PIC) method. J. Surfactants Deterg..

[108] Dua J.S., Rana A.C., Bhandari A.K. (2012). Liposome: Methods of preparation and applications. Int. J. Pharm. Sci. Rev..

[109] Pradhan B., Kumar N., Saha S., Roy A. (2015). Liposome: Method of preparation, advantages, evaluation and its application. J. Appl. Pharm. Res..

[110] Bangham A.D. (1978). Properties and uses of lipid vesicles: An overview. Ann. N. Y. Acad. Sci..

[111] Arabi M.H., Mirzapour A., Ardestani M.S., Saffari M. (2017). Preparation of nanoliposomes containing *Rosmarinus officinalis* L essential oil; A comparative study. Biosci. Biotechnol. Res. Commun..

[112] Varona S., Martin A., Cocero M.J. (2011). Liposomal incorporation of lavandin essential oil by a thin-film hydration method and by particles from gas-saturated solutions. Ind. Eng. Chem. Res..

[113] Noei F., Mirzaei H., Anarjan N., Javadi A., Behnajady M.A. (2025). Synthesis and characterization of nanoliposomes containing rosemary essential oil by thin-layer hydration-ultrasonication method: Preparation, optimization, and characterization. J. Food Meas. Charact..

[114] Whyms S.E., Nagar S., Luker H.A., Lopez A.D., Pigott M., Hodkinson T.R., Sheridan H., Hansen I.A., Walsh J.J. (2026). Repellent activity against *Aedes aegypti* and metabolomic profiling of *Myrica gale* L. essential oils from Irish boglands. Sci. Rep..

[115] Rahmana Putra N., Mamat H., Abdul Aziz A.H., Fatkhur Rozy M.I., Airlangga B. (2026). Cinnamon essential oil for food preservation: A comprehensive review of antimicrobial and antioxidant properties. J. Culin. Sci. Technol..

[116] Riaz M. (1996). Liposomes preparation methods. Pak. J. Pharm. Sci..

[117] Van Vuuren S.F., du Toit L.C., Parry A., Pillay V., Choonara Y.E. (2010). Encapsulation of Essential Oils within a Polymeric Liposomal Formulation for Enhancement of Antimicrobial Efficacy. Nat. Prod. Commun..

[118] Domazou A.S., Luigi Luisi P. (2002). Size distribution of spontaneously formed liposomes by the alcohol injection method. J. Liposome Res..

[119] Moghimipour E., Aghel N., Mahmoudabadi A.Z., Ramezani Z., Handali S. (2012). Preparation and characterization of liposomes containing essential oil of *Eucalyptus camaldulensis* leaf. Jundishapur J. Nat. Pharm. Prod..

[120] Wen Z., You X., Jiang L., Liu B., Zheng Z., Pu Y., Cheng B. (2011). Liposomal incorporation of rose essential oil by a supercritical process. Flavour Fragr. J..

[121] Castile J.D., Taylor K.M.G. (1999). Factors affecting the size distribution of liposomes produced by freeze–thaw extrusion. Int. J. Pharm..

[122] William B., Noemie P., Brigitte E., Geraldine P. (2020). Supercritical fluid methods: An alternative to conventional methods to prepare liposomes. Chem. Eng. J..

[123] Huang Z., Li X., Zhang T., Song Y., She Z., Li J., Deng Y. (2014). Progress involving new techniques for liposome preparation. Asian J. Pharm. Sci..

[124] Severino P., Santana M.H.A., Souto E.B. (2012). Optimizing SLN and NLC by 22 full factorial design: Effect of homogenization technique. Mater. Sci. Eng. C.

[125] Sastri K.T., Radha G.V., Pidikiti S., Vajjhala P. (2020). Solid lipid nanoparticles: Preparation techniques, their characterization, and an update on recent studies. J. Appl. Pharm. Sci..

[126] Fazly Bazzaz B.S., Khameneh B., Namazi N., Iranshahi M., Davoodi D., Golmohammadzadeh S. (2018). Solid lipid nanoparticles carrying *Eugenia caryophyllata* essential oil: The novel nanoparticulate systems with broad-spectrum antimicrobial activity. Lett. Appl. Microbiol..

[127] Tian H., Lu Z., Li D., Hu J. (2018). Preparation and characterization of citral-loaded solid lipid nanoparticles. Food Chem..

[128] Moghimipour E., Ramezani Z., Handali S. (2013). Solid lipid nanoparticles as a delivery system for *Zataria multiflora* essential oil: Formulation and characterization. Curr. Drug Deliv..

[129] Katopodi A., Detsi A. (2021). Solid Lipid Nanoparticles and Nanostructured Lipid Carriers of natural products as promising systems for their bioactivity enhancement: The case of essential oils and flavonoids. Colloids Surf. A Physicochem. Eng. Asp..

[130] Kelidari H.R., Moemenbellah-Fard M.D., Morteza-Semnani K., Amoozegar F., Shahriari-Namadi M., Saeedi M., Osanloo M. (2021). Solid-lipid nanoparticles (SLN) s containing *Zataria multiflora* essential oil with no-cytotoxicity and potent repellent activity against Anopheles stephensi. J. Parasit. Dis..

[131] Guterres S.S., Alves M.P., Pohlmann A.R. (2007). Polymeric nanoparticles, nanospheres and nanocapsules, for cutaneous applications. Drug Target Insights.

[132] Elmowafy M., Shalaby K., Elkomy M.H., Alsaidan O.A., Gomaa H.A.M., Abdelgawad M.A., Mostafa E.M. (2023). Polymeric Nanoparticles for Delivery of Natural Bioactive Agents: Recent Advances and Challenges. Polymers.

[133] Miladi K., Ibraheem D., Iqbal M., Sfar S., Fessi H., Elaissari A. (2014). Particles from preformed polymers as carriers for drug delivery. EXCLI J..

[134] Alvarez-Román R., Barré G., Guy R.H., Fessi H. (2001). Biodegradable polymer nanocapsules containing a sunscreen agent: Preparation and photoprotection. Eur. J. Pharm. Biopharm..

[135] Zielińska A., Carreiró F., Oliveira A.M., Neves A., Pires B., Venkatesh D.N., Durazzo A., Lucarini M., Eder P., Silva A.M. (2020). Polymeric Nanoparticles: Production, Characterization, Toxicology and Ecotoxicology. Molecules.

[136] Liakos I.L., Iordache F., Carzino R., Scarpellini A., Oneto M., Bianchini P., Grumezescu A.M., Holban A.M. (2018). Cellulose acetate-essential oil nanocapsules with antimicrobial activity for biomedical applications. Colloids Surf. B Biointerfaces.

[137] Ephrem E., Greige-Gerges H., Fessi H., Charcosset C. (2014). Optimisation of rosemary oil encapsulation in polycaprolactone and scale-up of the process. J. Microencapsul..

[138] Sotelo-Boyás M.E., Correa-Pacheco Z.N., Bautista-Baños S., Corona-Rangel M.L. (2017). Physicochemical characterization of chitosan nanoparticles and nanocapsules incorporated with lime essential oil and their antibacterial activity against food-borne pathogens. Food Sci. Technol..

[139] Lammari N., Louaer O., Meniai A.H., Elaissari A. (2020). Encapsulation of Essential Oils via Nanoprecipitation Process: Overview, Progress, Challenges and Prospects. Pharmaceutics.

[140] Vrouvaki I., Koutra E., Kornaros M., Avgoustakis K., Lamari F.N., Hatziantoniou S. (2020). Polymeric Nanoparticles of *Pistacia lentiscus* var. *chia* Essent. Oil Cutan. Applications. Pharmaceutics.

[141] Stancu A.I., Mititelu M., Ficai A., Ditu L.-M., Buleandră M., Badea I.A., Pincu E., Stoian M.C., Brîncoveanu O., Boldeiu A. (2025). Comparative evaluation of β-cyclodextrin inclusion complexes with eugenol, eucalyptol, and clove essential oil: Characterisation and antimicrobial activity assessment for pharmaceutical applications. Pharmaceutics.

[142] El Kharraf S., Farah A., El Hadrami E.M., El-Guendouz S., Lourenco J.P., Rosa Costa A.M., Miguel M.G. (2021). Encapsulation of *Rosmarinus officinalis* essential oil in β-cyclodextrins. J. Food Process. Preserv..

[143] Waleczek K.J., Marques H.M., Hempel B., Schmidt P.C. (2003). Phase solubility studies of pure (-)-alpha-bisabolol and camomile essential oil with beta-cyclodextrin. Eur. J. Pharm. Biopharm..

[144] Castro J.C., Pante G.C., de Souza D.S., Pires T.Y., Miyoshi J.H., Garcia F.P., Nakamura C.V., Mulati A.C.N., Mossini S.A.G., Junior M.M. (2022). Molecular inclusion of *Cymbopogon martinii* essential oil with β-cyclodextrin as a strategy to stabilize and increase its bioactivity. Food Hydrocoll. Health.

[145] Cetin Babaoglu H., Bayrak A., Ozdemir N., Ozgun N. (2017). Encapsulation of clove essential oil in hydroxypropyl beta-cyclodextrin for characterization, controlled release, and antioxidant activity. J. Food Process. Preserv..

[146] Galvão J.G., Cerpe P., Santos D.A., Gonsalves J.K.M.C., Santos A.J., Nunes R.K.V., Lira A.A.M., Alves P.B., La Corte R., Blank A.F. (2019). *Lippia gracilis* essential oil in β-cyclodextrin inclusion complexes: An environmentally safe formulation to control *Aedes aegypti* larvae. Pest. Manag. Sci..

[147] Mendes L.A., Silva R.R.A., Soares N.d.F.F., Martins G.F., Teixeira R.R., da Silva Ferreira M.F., Moreira R.P.L. (2023). Development of inclusion complexes of 2-hydroxypropyl-β-cyclodextrin with *Psidium guajava* L. essential oil by freeze-drying and kneading methods for application as *Aedes aegypti* L. larvicide. Nat. Prod. Res..

[148] Loftsson T., Jarho P., Másson M., Järvinen T. (2005). Cyclodextrins in drug delivery. Expert Opin. Drug Deliv..

[149] Enciso-Sáenz S., Borrás-Enriquez A.J., Ventura-Canseco L.M.C., Gutiérrez-Miceli F., Dendooven L., Grajales-Lagunes A., Ruiz-Cabrera M.A., Ruíz-Valdiviezo V., Abud-Archila M. (2018). Lemongrass (*Cymbopogon citratus* (DC) Stapf) essential oil encapsulation by freeze-drying. Rev. Mex. De Ing. Quím..

[150] Setzer W.N., Satyal P. (2026). Cedarwood Oils: The Wood Essential Oil Compositions from Trees Known as “Cedar”. Plants.

[151] van Beek T.A., Joulain D. (2018). The essential oil of patchouli, *Pogostemon cablin*: A review. Flavour Fragr. J..

[152] Seibert J.B., Rodrigues I.V., Carneiro S.P., Amparo T.R., Lanza J.S., Frézard F.J.G., de Souza G.H.B., dos Santos O.D.H. (2019). Seasonality study of essential oil from leaves of *Cymbopogon densiflorus* and nanoemulsion development with antioxidant activity. Flavour Fragr. J..

[153] Taleb M.H., Abdeltawab N.F., Shamma R.N., Abdelgayed S.S., Mohamed S.S., Farag M.A., Ramadan M.A. (2018). *Origanum vulgare* L. essential oil as a potential anti-acne topical nanoemulsion—In vitro and in vivo study. Molecules.

[154] Mozafari M.R. (2005). Liposomes: An overview of manufacturing techniques. Cell. Mol. Biol. Lett..

[155] Sercombe L., Veerati T., Moheimani F., Wu S.Y., Sood A.K., Hua S. (2015). Advances and Challenges of Liposome Assisted Drug Delivery. Front. Pharmacol..

[156] Michel R., Plostica T., Abezgauz L., Danino D., Gradzielski M. (2013). Control of the stability and structure of liposomes by means of nanoparticles. Soft Matter.

[157] Mohammed A.R., Bramwell V.W., Coombes A.G.A., Perrie Y. (2006). Lyophilisation and sterilisation of liposomal vaccines to produce stable and sterile products. Methods.

[158] Grit M., Crommelin D.J.A. (1993). Chemical stability of liposomes: Implications for their physical stability. Chem. Phys. Lipids.

[159] Aslan M., Ertaş N., Demir M.K. (2023). Storage stability, heat stability, controlled release and antifungal activity of liposomes as alternative fungal preservation agents. Food Biosci..

[160] Silva E.F.D., Santos F., Pires H.M., Bastos L.M., Ribeiro L.N.M. (2025). Lipid Nanoparticles Carrying Essential Oils for Multiple Applications as Antimicrobials. Pharmaceutics.

[161] Cimino C., Maurel O.M., Musumeci T., Bonaccorso A., Drago F., Souto E.M.B., Pignatello R., Carbone C. (2021). Essential Oils: Pharmaceutical Applications and Encapsulation Strategies into Lipid-Based Delivery Systems. Pharmaceutics.

[162] Saporito F., Sandri G., Bonferoni M.C., Rossi S., Boselli C., Icaro Cornaglia A., Mannucci B., Grisoli P., Vigani B., Ferrari F. (2018). Essential oil-loaded lipid nanoparticles for wound healing. Int. J. Nanomed..

[163] Souza P.H.O., Uchôa A.F.C., Formiga A.L.D., Carvalho L.M.M., Pereira G.M.A., Silva L.F.A., Passos M.F., Barbosa-Filho J.M., Silva M.S., Xavier-Júnior F.H. (2026). Process optimization and stability enhancement of *Origanum vulgare* L. essential oil-loaded nanostructured lipid carriers. Appl. Food Res..

[164] Romero-Montero A., Medel E., Gutiérrez-Flores J., García-Aguirre I., Vargas R., Del Prado-Audelo M.L. (2026). The Crucial Role of Noncovalent Interactions in PLGA Nanoparticles: Impact on Carvacrol/p-Cymene Release, Biological Activity, and Antibiofilm Properties. Am. Chem. Soc. Omega.

[165] Martins I.M., Rodrigues S.N., Barreiro M.F., Rodrigues A.E. (2012). Release Studies of Thymol and p-Cymene from Polylactide Microcapsules. Ind. Eng. Chem. Res..

[166] dos Passos Menezes P., Serafini M.R., de Carvalho Y.M.B.G., Santana D.V.S., Lima B.S., Quintans-Júnior L.J., Marreto R.N., de Aquino T.M., Sabino A.R., Scotti L. (2016). Kinetic and physical-chemical study of the inclusion complex of β-cyclodextrin containing carvacrol. J. Mol. Struct..

[167] Nieddu M., Rassu G., Boatto G., Bosi P., Trevisi P., Giunchedi P., Carta A., Gavini E. (2014). Improvement of thymol properties by complexation with cyclodextrins: In vitro and in vivo studies. Carbohydr. Polym..

[168] Costa M.d.S., Rocha J.E., Campina F.F., Silva A.R.P., Da Cruz R.P., Pereira R.L.S., Quintans-Júnior L.J., De Menezes I.R.A., Araújo A.A.d.S., De Freitas T.S. (2019). Comparative analysis of the antibacterial and drug-modulatory effect of d-limonene alone and complexed with β-cyclodextrin. Eur. J. Pharm. Sci..

[169] Gong L., Li T., Chen F., Duan X., Yuan Y., Zhang D., Jiang Y. (2016). An inclusion complex of eugenol into β-cyclodextrin: Preparation, and physicochemical and antifungal characterization. Food Chem..

[170] Hedges A.R. (2009). Cyclodextrins: Properties and Applications. Food Sci. Technol..

[171] Janadri S., Manjunatha P., Sharma U.R., Vada S., Haribabu T., Madhu M., Angadi P.P. (2025). A Systemic Review of Beta Caryophyllene. Def. Life Sci. J..

[172] Connors K.A. (1997). The stability of cyclodextrin complexes in solution. Chem. Rev..

[173] Loftsson T., Masson M. (2001). Cyclodextrins in topical drug formulations: Theory and practice. Int. J. Pharm..

[174] Chang Y., McLandsborough L., McClements D.J. (2013). Physicochemical properties and antimicrobial efficacy of carvacrol nanoemulsions formed by spontaneous emulsification. J. Agric. Food Chem..

[175] Salvia-Trujillo L., Rojas-Graü A., Soliva-Fortuny R., Martín-Belloso O. (2015). Physicochemical characterization and antimicrobial activity of food-grade emulsions and nanoemulsions incorporating essential oils. Food Hydrocoll..

[176] Tadros T., Izquierdo P., Esquena J., Solans C. (2004). Formation and stability of nano-emulsions. Adv. Colloid Interface Sci..

[177] Clavijo-Romero A., Quintanilla-Carvajal M.X., Ruiz Y. (2019). Stability and antimicrobial activity of eucalyptus essential oil emulsions. Food Sci. Technol. Int..

[178] Ortan A. (2009). Studies concerning the entrapment of *Anethum graveolens* essential oil in liposomes. Roum. Biotechnol. Lett..

[179] Liolios C.C., Gortzi O., Lalas S., Tsaknis J., Chinou I. (2009). Liposomal incorporation of carvacrol and thymol isolated from the essential oil of *Origanum dictamnus* L. and in vitro antimicrobial activity. Food Chem..

[180] Ghodrati M., Farahpour M.R., Hamishehkar H. (2019). Encapsulation of Peppermint essential oil in nanostructured lipid carriers: In-vitro antibacterial activity and accelerative effect on infected wound healing. Colloids Surf. A Physicochem. Eng. Asp..

[181] Pirouzifard M.K., Hamishehkar H., Pirsa S. (2020). Cocoa butter and cocoa butter substitute as a lipid carrier of *Cuminum cyminum* L. essential oil; physicochemical properties, physical stability and controlled release study. J. Mol. Liq..

[182] Khezri K., Farahpour M.R., Mounesi Rad S. (2019). Accelerated infected wound healing by topical application of encapsulated rosemary essential oil into nanostructured lipid carriers. Artif. Cells Nanomed. Biotechnol..

[183] Khezri K., Farahpour M.R., Mounesi Rad S. (2020). Efficacy of *Mentha pulegium* essential oil encapsulated into nanostructured lipid carriers as an in vitro antibacterial and infected wound healing agent. Colloids Surf. A Physicochem. Eng. Asp..

[184] Froiio F., Ginot L., Paolino D., Lebaz N., Bentaher A., Fessi H., Elaissari A. (2019). Essential Oils-Loaded Polymer Particles: Preparation, Characterization and Antimicrobial Property. Polymers.

[185] Choudhary A., Salar R.K., Thakur R. (2024). Synthesis, characterization and insecticidal activity of *Mentha spicata* essential oil loaded polymeric nanoparticles. Biocatal. Agric. Biotechnol..

[186] Feyzioglu G.C., Tornuk F. (2016). Development of chitosan nanoparticles loaded with summer savory (*Satureja hortensis* L.) essential oil for antimicrobial and antioxidant delivery applications. Food Sci. Technol..

[187] Ahmadi Z., Saber M., Akbari A., Mahdavinia G.R. (2018). Encapsulation of *Satureja hortensis* L. (Lamiaceae) in chitosan/TPP nanoparticles with enhanced acaricide activity against *Tetranychus urticae* Koch (Acari: Tetranychidae). Ecotoxicol. Environ. Saf..

[188] Yeguerman C.A., Urrutia R.I., Jesser E.N., Massiris M., Delrieux C.A., Murray A.P., González J.O.W. (2022). Essential oils loaded on polymeric nanoparticles: Bioefficacy against economic and medical insect pests and risk evaluation on terrestrial and aquatic non-target organisms. Environ. Sci. Pollut. Res..

[189] da Rocha Neto A.C., de Oliveira da Rocha A.B., Maraschin M., Di Piero R.M., Almenar E. (2018). Factors affecting the entrapment efficiency of β-cyclodextrins and their effects on the formation of inclusion complexes containing essential oils. Food Hydrocoll..

[190] Ayala-Zavala J.F., Soto-Valdez H., González-León A., Álvarez-Parrilla E., Martín-Belloso O., González-Aguilar G.A. (2008). Microencapsulation of cinnamon leaf (*Cinnamomum zeylanicum*) and garlic (*Allium sativum*) oils in β-cyclodextrin. J. Incl. Phenom. Macrocycl. Chem..

[191] de Freitas C.A.B., Costa C.H.S., da Costa K.S., da Paz S.P.A., Silva J.R.A., Alves C.N., Lameira J. (2023). Assessment of host–guest molecular encapsulation of eugenol using β-cyclodextrin. Front. Chem..

[192] Lin J., Wu K., Wang P., Duan X., Chen J., Ding T., Xue H. (2025). Rational cyclodextrin selection for cinnamon essential oil encapsulation: Performance regulation based on physicochemical characterization and molecular simulation. Food Chem..

[193] Guo J., Jiang X., Tian Y., Yan S., Liu J., Xie J., Zhang F., Yao C., Hao E. (2024). Therapeutic potential of cinnamon oil: Chemical composition, pharmacological actions, and applications. Pharmaceuticals.

[194] Hyldgaard M., Mygind T., Meyer R.L. (2012). Essential Oils in Food Preservation: Mode of Action, Synergies, and Interactions with Food Matrix Components. Front. Microbiol..

[195] Sun J., Leng X., Zang J., Zhao G. (2024). Bio-based antibacterial food packaging films and coatings containing cinnamaldehyde: A review. Crit. Rev. Food Sci. Nutr..

[196] Zhou L., Cai Y., Li Y., Zhou Y., Xiao Z. (2025). Cinnamon essential oil delivery systems: Preparation processes, packaging forms, and industrialization potentials. Food Sci. Nutr..

[197] Chen J., Li S., Zheng Q., Feng X., Tan W., Feng K., Liu Y., Hu W. (2022). Preparation of solid lipid nanoparticles of cinnamaldehyde and determination of sustained release capacity. Nanomaterials.

[198] Burt S. (2004). Essential oils: Their antibacterial properties and potential applications in foods—A review. Int. J. Food Microbiol..

[199] Jopa S., Wójcik J., Ejchart A., Nowakowski M. (2024). NMR methods for studying inclusion complexes focused on chiral hosts. J. Incl. Phenom. Macrocycl. Chem..

[200] Guidotti-Takeuchi M., de Morais Ribeiro L.N., dos Santos F.A.L., Rossi D.A., Lucia F.D., de Melo R.T. (2022). Essential Oil-Based Nanoparticles as Antimicrobial Agents in the Food Industry. Microorganisms.

[201] Guimarães A.C., Meireles L.M., Lemos M.F., Guimarães M.C.C., Endringer D.C., Fronza M., Scherer R. (2019). Antibacterial Activity of Terpenes and Terpenoids Present in Essential Oils. Molecules.

[202] Mears K.L., Kutzleb M.A., Stennett C.R., Fettinger J.C., Kaseman D.C., Yu P., Vasko P., Power P.P. (2022). Terpene dispersion energy donor ligands in borane complexes. Chem. Commun..

[203] Grimme S., Schreiner P.R. (2024). The role of london dispersion interactions in modern chemistry. Acc. Chem. Res..

[204] Griffin S.G., Wyllie S.G., Markham J.L., Leach D.N. (1999). The role of structure and molecular properties of terpenoids in determining their antimicrobial activity. Flavour Fragr. J..

[205] Keiluweit M., Kleber M. (2009). Molecular-level interactions in soils and sediments: The role of aromatic π-systems. Environ. Sci. Technol..

[206] Kfoury M., Auezova L., Ruellan S., Greige-Gerges H., Fourmentin S. (2015). Complexation of estragole as pure compound and as main component of basil and tarragon essential oils with cyclodextrins. Carbohydr. Polym..

[207] Lémery E., Briançon S., Chevalier Y., Bordes C., Oddos T., Gohier A., Bolzinger M.-A. (2015). Skin toxicity of surfactants: Structure/toxicity relationships. Colloids Surf. A Physicochem. Eng. Asp..

[208] Yang H., Li R., Ma H., Tian L., Yan J., Chen G., Zhang Z. (2025). Development of *Forsythia* essential oil microemulsions: Effects of surfactants on stability and antibacterial activity. ACS Omega.

[209] Ibrahim S.S., Gondhalekar A.D., Ristroph K., Baributsa D. (2025). Geranium Oil Nanoemulsion Delivers More Potent and Persistent Fumigant Control of *Callosobruchus maculatus* in Stored Grain. Foods.

[210] Danhier F., Lecouturier N., Vroman B., Jérôme C., Marchand-Brynaert J., Feron O., Préat V. (2009). Paclitaxel-loaded PEGylated PLGA-based nanoparticles: In vitro and in vivo evaluation. J. Control. Release.

[211] Stella V.J., He Q. (2008). Cyclodextrins. Toxicol. Pathol..

[212] Thomas T., Thomas K., Sadrieh N., Savage N., Adair P., Bronaugh R. (2006). Research strategies for safety evaluation of nanomaterials, part VII: Evaluating consumer exposure to nanoscale materials. Toxicol. Sci..

[213] Buzea C., Pacheco I.I., Robbie K. (2007). Nanomaterials and nanoparticles: Sources and toxicity. Biointerphases.

[214] Ranjan S., Dasgupta N., Singh S., Gandhi M. (2019). Toxicity and regulations of food nanomaterials. Environ. Chem. Lett..

